# Diurnal light fitness of the C3 and C4 species from the genus *Atriplex* under control and drought conditions

**DOI:** 10.1007/s11120-025-01154-5

**Published:** 2025-06-11

**Authors:** Reham M. Nada, Abdel Hamid A. Khedr, Mamdouh S. Serag, Nesma R. El-Qashlan, Gaber M. Abogadallah

**Affiliations:** Department of Botany and Microbiology, Faculty of Science, Damietta University, New Damietta, 34517 Egypt

**Keywords:** *Atriplex*, C4 species, C3 species, Light intensity, Drought

## Abstract

The literature showed contradictory results regarding the acclimation of C3 and C4 photosynthesis to low light intensities. *Atriplex halimus*,* A. nummularia* (C4, NAD-ME), *A. portulacoides* and *A. prostrata* (C3) were exposed to three natural light intensities: full light (FL), medium light (ML) and low light (LL) under control or drought condition. Under control condition, in *A. halimus* and *A. nummularia*, photosynthetic rate (*A*) was proportionally linked to stomatal conductance (*g*_*s*_). In *A. halimus*, *A* and *gs* peaked at 9:00 and 12:00 at FL only. However, *A* and *gs* peaked at 9:00 and 12:00 under FL and ML, respectively, in *A. nummularia*. The leakage of CO_2_ could limit *A* in the C4 species under lower light intensities. *A. halimus* reduced *g*_*s*_ and *A* (a typical NAD-ME strategy) to cope with lower light intensities. However, *A. nummularia* optimized leaf anatomical features and PEPC/ Rubisco ratio to reduce CO_2_ leakage, leading to improved *g*_*s*_, *A* and biomass. In contrast, the increase in *g*_*s*_ reflected no increase in *A*, which could be attributed to the negative effect of low light on the electron transport system in the C3 species. Under drought condition, the performance of the C3 and C4 species was better at ML and LL than that at FL because of enhanced *g*_*s*_ and *A*. The present study concluded that the C4 species acclimated better to low light intensities than the C3 species. The acclimation of the C4 species was dependent on the species and the soil water content rather than the biochemical subtype.

**Supplementary Information:**

The online version contains supplementary material available at 10.1007/s11120-025-01154-5.

## Introduction

The physiological differences between the C3 and C4 photosynthetic pathways provide the major pattern of the ecology and evolution of the C3 and C4 plants, where the two pathways translate into distinct physiological advantages and disadvantages under different environmental conditions (Berry 1975; Ehleringer and Bjorkman 1977). Light is one of the most dynamic environmental factors that affect plant performance (Ruban 2015). The evolution of C4 plants was to develop mechanisms that make these plants tolerate harsh conditions -including high light intensity- compared to their C3 counterparts (Osborne and Sack 2012). The CO_2_ concentrating mechanism in C4 leaves depends on two distinct cell types: mesophyll and bundle sheath cells. According to the reaction pathways (reviewed in full detail in Yin and Struik 2018), extra ATP is required for the CO_2_ concentrating mechanism in addition to that required for the C3 cycle. Therefore, the fixation rate of CO_2_ is limited under low light intensity by the electron transport rate and the need for high ATP consumption in the bundle sheath cells in the C4 plants (Tazoe et al. 2008). Therefore, Li et al. (2021) concluded that C4 plants have lower light-utilization efficiency than C3 ones under fluctuating light. In contrast, Cubas et al. (2023) found that the assimilation rate of the C4 species was stronger and more sustained than that of the C3 species under the low light phase of fluctuating light conditions.

The response of C3 and C4 plants to low light irradiance involves the acclimation of photosynthetic apparatus to maximize the light use efficiency for proper CO_2_ carboxylation (Sage and McKown 2006). Acclimation of C3 and C4 to shade involves the partitioning of photosynthetic nitrogen away from Rubisco to light-harvesting systems (Boardman 1977; Evans and Poorter 2001; Pengelly et al. 2010). C4 plants are categorized into three biochemical subtypes: NADP-malic enzyme, NAD-ME and phosphoenolpyruvate carboxykinase (PEP-CK) according to the decarboxylation enzymes (Hatch 1987) and they differently respond to low light intensity (Sonawane et al. 2018). C4 NAD-ME species have higher leaf N content and N fraction invested in Rubisco compared to C4 NADP-ME plants (Ghannoum et al. 2005). Therefore, C4 NAD-ME plants may be more flexible to reallocate N under low light intensity and thus reducing the activity of the C3 cycle more than the C4 cycle. However, the optimal acclimation to shade is expected to be a parallel reduction in C3 and C4 cycle in order to cope with CO_2_ leakiness and maintain an efficient CO_2_ concentration mechanism and photosynthetic rate (Bellasio and Griffiths 2014a, b), which is not the case of C4 NAD-ME species (Buchmann et al. 1996). Therefore, Sonawane et al. (2018) concluded that C4 NAD-ME species could acclimate to low light irradiance by a greater photosynthetic downregulation and higher CO_2_ leakiness compared to the other two subtypes of C4 species. However, we hypothesized that this conclusion may not be a general one where the response to different light intensities could depend on species (even having the same biochemical subtype) rather than on the biochemical subtypes. Cubas et al. (2023) suggested that the pattern of assimilation rate was dependent on species or C4 subtypes rather than photosynthetic pathway at the phase of high light during fluctuating light conditions.

Typically, stomatal pores in the C3 and C4 species open in response to light, low CO_2_ concentration and high humidity, whereas the closure is regulated by darkness, high CO_2_, low humidity and high temperature (Lawson et al. 2011 and ref. therein). Most studies have demonstrated that stomatal conductance declined in response to low light intensity treatment in C3 species (e.g. Zhang et al. 2016; Tang et al. 2022), leading to a lower photosynthetic rate. In C4 species, the closure of stomata may not be the main factor that negatively affects the photosynthetic rate since there are other parameters that could be involved, such as bundle sheath leakiness, leading to the imbalance between the C4 and C3 cycle (Kubásek et al. 2013). However, in both C3 and C4 species, increased stomatal conductance could remove the stomatal limitation on photosynthesis and enhance carbon uptake, whereas decreased stomatal conductance (especially under soil water limitation) could enhance the water use efficiency but at the expense of the assimilation rate (Jones 1987). It has been reported that removal of the stomatal limitation could enhance the photosynthetic rate by 10–20% in the C3 plants and even less in the C4 plants (Farquhar and Sharkey 1982; Jones 1985). In the present study, we hypothesized that the effect of the removal of stomatal limitation on carbon gain could be dependent on the soil water content and plant species rather than photosynthesis type or biochemical subtype.

Drought stress is also one of the main environmental factors that affect plant growth and productivity in the C3 and C4 plants. Drought stress leads to a reduction in the water content of cells that resulted in a decline in stomatal conductance, photosynthetic rate, growth and productivity (Li et al. 2017; Jumrani and Bhatia 2018; Castro et al. 2019). However, the C4 plants can withstand drought stress compared to the C3 ones (Hura et al. 2007). Also, it has been known that the C4 species are more water-use efficient than the C3 ones (Way et al. 2014 and ref. therein). Little is known about how different light intensities modulate the response of plants to drought stress. Combined drought stress and excess light could severely reduce plant growth (Obermeyer 2017). In contrast, shading could alleviate the negative impact of drought stress by reducing the temperature, water pressure deficit and oxidative stress (Holmgren 2000).

Light stress (e.g. Noguchi et al. 2005; Yoshida et al. 2008; Zhu et al. 2017) or drought stress (Wang et al. 2015; Rivas et al. 2017) negatively affects the photosynthetic system, which may lead to oxidative stress. It has been suggested that *AOX* plays an essential role in preventing over-reduction of chloroplast by efficient dissipation of excess cellular reducing equivalents (Raghavendra and Padmasree 2003; McDonald 2008). *AOX* may function as a sink for excess equivalents generated in the chloroplast (Xu et al. 2011). At photosystem I, ferredoxin NADP^+^ reductase (FNR) receives electrons from the reduction of ferredoxin at the end of the electron transport chain and converts NADP^+^ to NADPH (Shin et al. 1963; Kimata-Ariga et al. 2019), which is required for CO_2_ fixation. Overexpression of FNR was effective in increasing plant tolerance to oxidative and high light stresses (Rodriguez-Heredia et al. 2022). In the present study, *AOX* and *FNR* were chosen to determine their role in the combined effect of different light intensities and drought stress.

The present study was carried out because of: 1- the contrasting results obtained from studying the response of C3 and C4 plants to low light intensities, 2- most of these studies investigated the response of the C4 species under synthesized fluctuating light conditions at fixed time points but not at diurnal rhythm under natural conditions, 3- most of the previous studies were concerned with studying the effect of different light intensities and drought stress on crop species because of their economic importance. However, studying the wild species that endure and tolerate different environmental factors may lead to the identification of the mechanisms underpinning their response, 4- plenty of studies have separately investigated the response of C3 or C4 species to different light intensities in the presence of drought stress and some of them had contradictory results about the response of these species to different light intensities. Comparing the light fitness of the C4 and C3 may support the development of improvement strategies for crops, including the engineering of C4 photosynthesis into C3 plants (Ermakova et al., 2021).

In the present study, we focused on four species belonging to the genus *Atriplex*; two of them are C3 (*Atriplex portulacoides* and *Atriplex prostrata*) and the other two are C4 species (*Atriplex halimus* and *Atriplex nummularia*). Both *A. halimus* and *A. nummularia* are from NAD-ME subgroup (Sage 2003). Genus *Atriplex* is dominant in arid and semi-arid habitats and also it dominates areas that combine aridity with high soil salinity or water shortage (Osmond et al. 1980; McArthur and Sanderson 1984). *Atriplex* species are known to have the ability to complete their life cycle under harsh conditions such as drought, salinity or high temperature (Ramos et al., 2004). Although salt and drought tolerance of *Atriplex* species was heavily studied, the response of genus *Atriplex* (C3 and C4) to different light intensities, particularly to low light irradiance is not fully elucidated. The present study aimed at answering the following questions: Does C4 spp. of *Atriplex* tolerate low light intensity? Do both *A. halimus* and *A. nummularia* (both from NAD-ME subgroup) follow a similar manner in their response to low light intensity? Does the photosynthetic performance of the C3 spp. of *Atriplex* show efficient regulation under low light intensity compared to the C4 spp.? Does low light intensity enhance the performance of photosynthesis and growth of the C3 and C4 spp. under drought stress?

The objectives of the present study were to 1- investigate the diurnal regulation of photosynthesis of the C3 and C4 spp. at different light intensities under control and drought conditions, 2- determine the factors (external or internal) regulating the photosynthetic and growth performance at low light intensity under control and drought conditions.

## Materials and methods

### Experimental design

The present study focused on four species from the genus *Atriplex*; two follow C3 photosynthesis (*Atriplex portulacoides* and *A. prostrata)* and the other two follow C4 photosynthesis (*A. halimus* and *A. nummularia)*. After several surveys, homogenous seedlings of the four *Atriplex* species were selected and transplanted from their natural habitat to the greenhouse of the Botany and Microbiology Department, Faculty of Science, Damietta University (Fig. S1). The transplanted seedlings were left to acclimate to the greenhouse conditions until new leaves were evolved. After the acclimation period, the experimental layout was designed as follows. The seedlings were distributed into six blocks; three blocks were used for the control (control group) treatment and the other three blocks were used for the drought treatment (drought group). Each block contained the four species. These seedlings were irrigated daily. The three blocks of the control and the three blocks of the drought groups were subjected to three different light intensities (natural sunlight was used), where the first block of each treatment was exposed to 1221 µmol m^− 2^s^− 1^ maximum light intensity (full light intensity), the second block of each treatment was covered with wire mesh to let about 66% of the full sunlight (about 723 µmol m^− 2^ s^− 1^ light intensity; medium light intensity) and the third block of each treatment was covered with doubled-wire mesh to let about 33% of the full sunlight to pass (about 330 µmol m^− 2^ s^− 1^ light intensity; low light intensity). The light intensity was measured using a light meter at each point. Therefore, there were control with full light intensity, control with medium light intensity and control with low light intensity blocks and the same for the drought treatment. The drought treatment was applied for ten days. Therefore, each of the four *Atriplex* species was affected by two factors: different light intensities (full, medium and low) and different water regimes (control and drought). The climatic conditions over the experiment period were 32–35/27–30ºC day/night temperature and 39–41% relative humidity (RH) during the day. The field capacity was kept constant for the drought group over the treatment period (10 days). Field capacity (FC) was determined as follows: about 10 g of soil collected from the rhizosphere of each species from each treatment was saturated with water for 48 h and weighed to calculate the FC. The FC of the soil was calculated according to the equation in which the soil weight before saturation was divided by the weight of saturated soil. Unlike other studies (controlled and monitored conditions), this experiment was designed to mimic what happens at the natural conditions. The subsequent analyses were carried out at a diurnal pattern (9:00, 12:00 and 15:00), but the most pronounced and leading results were found at 9:00 and 12:00. After repeated examination all over the year, the experiment was carried out under conditions that let the plants (the C3 and C4) achieve optimal growth (the summer season for both photosynthetic types).

### Plant sampling

After 10 days after the drought treatment onset, the third leaf from the top of the four species from each treatment was collected in pre-weighted bags and used to determine fresh (FW) and dry (DW) weights and water content. Five replicates were used for each treatment. To measure specific leaf area (SLA), excised leaves were collected in pre-weighed plastic bags and weighed for fresh weight and then for dry weight after drying in an oven. Leaf area was determined by using Photoshop V 6.0 and then SLA was calculated by dividing leaf area by leaf dry weight. Five replicates were used for each plant for each treatment. Also, the third leaf from the top was collected, wrapped in aluminum foil, frozen immediately in liquid nitrogen and stored at -80 °C until used for the subsequent biochemical and molecular analyses. Ten leaves (replicates) from each plant from each treatment were collected at 9:00 and 12:00.

### Leaf sectioning to determine the change of anatomical features in response to each treatment

Leaf samples were collected and treated for the anatomical section as described in Johansen (1940) and modified by Abogadallah and Nada (2019). Five replicates were used for each treatment.

### Estimation of gas exchange parameters in response to different treatments (water regimes and light intensities)

Gas exchange parameters were measured at 9:00 and 12:00 for the third leaf from the top by using LCi-SD gas exchange system (Analytical Development Company Ltd., England). Photosynthetic rate (*A*), transpiration rate (*E*), stomatal conductance (*g*_*s*_), internal CO_2_ (*C*_*i*_), leaf temperature (*T*_*l*_) and quantum leaf (Q) were measured. Each leaf was equilibrated inside the leaf chamber for about 5 min until constant reading was recorded. Because of number of samples, the measurements were carried out over two days (temperature and RH within the range described above). Five readings were recorded for five different leaves from different plants of each species for each treatment.

### Determination of total soluble sugars (TSS) and starch contents

Total soluble sugars were extracted according to methods described in Schluter and Crawford (2001) and modified by Nada and Abogadallah (2014). Three replicates from three independent plants were used for each treatment.

### Determination of leaf pigments

Chlorophyll content was measured colorimetrically after extracting about 100 mg of freshly collected leaves by using 80% acetone according to methods described by Arnon (1949). Three replicates from three independent plants for each treatment were used.

### Quantification of Rubisco and PEPC proteins using SDS-PAGE

Leaf-soluble proteins were extracted by grinding 50 mg of the frozen leaves in 50 mM Hepes buffer (pH 7.4) containing 5 mM β-mercaptoethanol and 5 mM PMSF. The debris was removed by centrifugation at 12,000 rpm for 10 min at 4ºC. The protein concentration in the extracts was determined as described by Bradford (1976). The proteins were separated as described by Laemmli (1970) using the BioRad Mini Protean 3 (BioRad Laboratories, Hercules, CA, USA). The resolving gel contained 11% acrylamide and the stacking gel contained 5% acrylamide. About 100 µg protein was loaded onto each lane. The gels were stained with brilliant blue R-250 and then de-stained with 20% methanol. The gels were dried, scanned and used for Rubisco and PEPC quantification using Image Studio v3.1.4 software (Nada and Abogadallah 2014, 2016; Nada et al. 2018). Three replicates from three independent plants were used for each treatment.

### Quantification of gene expression by Semi-Quantitative RT-PCR (SQ-RT-PCR)

RNA was extracted from 50 mg of the frozen leaf samples by using total RNA mini kit according to the manufacture’s protocol (Cat. No. RP050, Geneaid Company, Taiwan). The extracted RNA was treated with DNase (DNA free kit, Cat. No. AM1906, Ambion, US) to remove DNA contamination according to the manufacturer’s protocol. cDNA was synthesized according to the protocol described in cDNA synthesis kit (Cat. No. RT220, enzynomics company, Korea). Primers for *AOX* and *FNR* genes were designed as degenerate primers to recognize the conserved motifs resulting from the alignment of the characterized protein sequences from other species found in NCBI and ExPASy databases. These genes were not characterized in any species of genus *Atriplex*; therefore, degenerate primers were designed. However, to reduce the degeneracy, primers were further re-designed based on the alignment of codon sequences identified in the other species (Table S1). We could not overcome dimers; therefore, SQ-RT-PCR was used to quantify the expression pattern of the genes. The PCR conditions were adjusted as follows: initial denaturation at 94 ^o^C for 3 min, followed by 35 cycles of denaturation at 94 ^o^C for 30 s, annealing at 50 ^o^C for 30 s and extension at 72 ^o^C for 45 s and then final extension at 72 ^o^C for 5 min. The number of cycles, annealing temperature and extension time were adjusted according to each primer pair for each gene. For each gene, the number of PCR cycles was optimized to show the maximum differences among samples within the linear phase of amplification. The conditions and cycles were adjusted to avoid DNA saturation. The PCR products were resolved on 1% agarose gel stained with EtBr in 1X TAE buffer using BioRad equipment and visualized by the gel documentation system. The band volume was measured by Image Studio v 3.1.4 software. Normalization of the measurements was carried out for equal *18S rRNA*, which was used as an internal control. Three replicates from three independent plants were used for each treatment.

### Statistical analysis

All measured parameters were replicated as mentioned in each section above. To compare within and among species under the combined treatments (light intensity and water regimes), MANOVA (one, two and three-way ANOVA) test with Fisher’s least significant difference (LSD) post hoc test was performed by using Sigma Plot v 11.0 at a significant level of *P* ≤ 0.05. The normality of results was tested by using Sigma Plot v 11.0.

## Results

### Leaf biomass and specific leaf area (SLA) at different light intensities under control and drought conditions

**Fig. 1:**
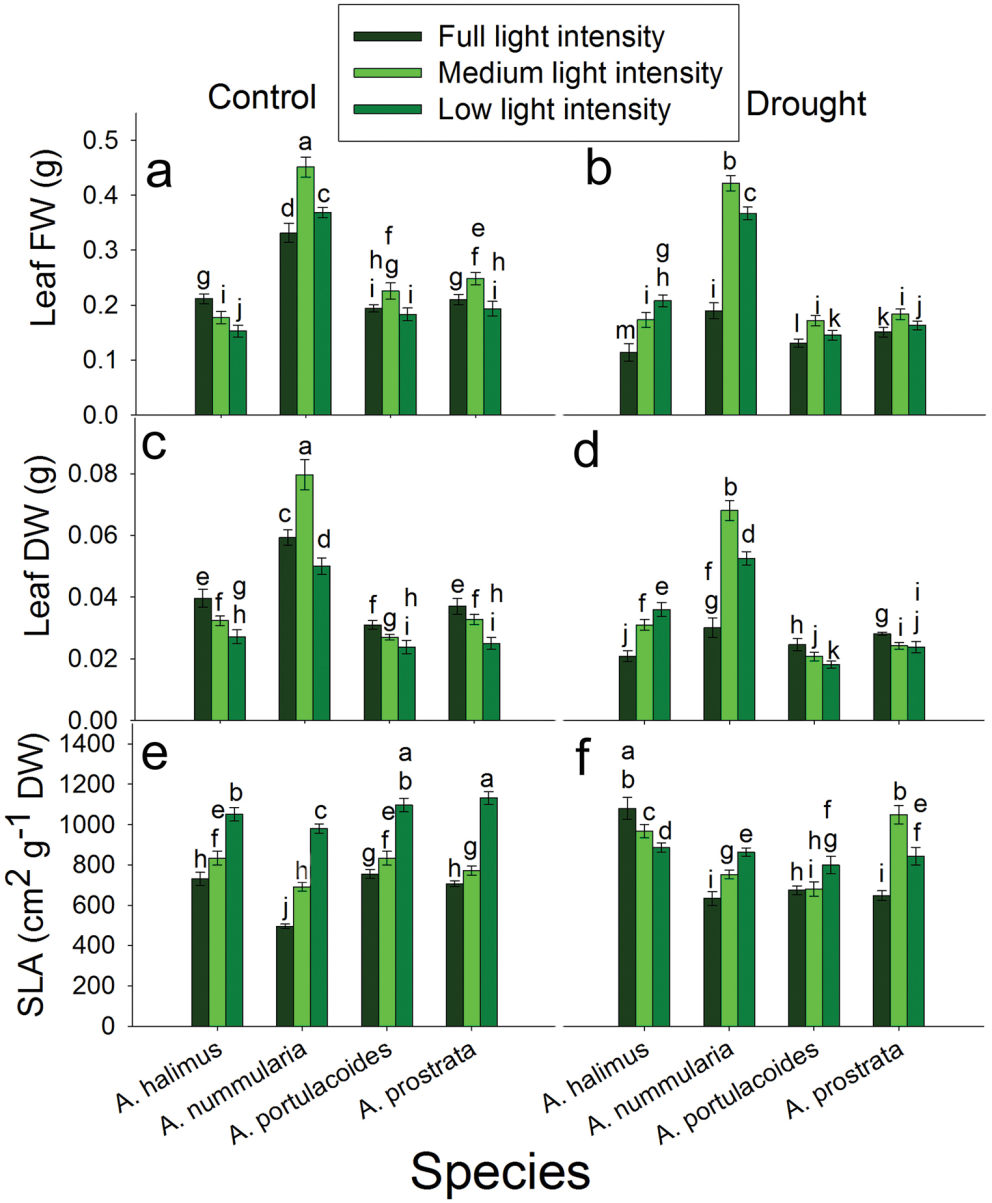
Effect of different light intensities on leaf biomass under control and drought conditions. **a** and **b**: leaf fresh weight under control and drought conditions, respectively. **c** and **d**: leaf dry weight under control and drought conditions, respectively. **e** and **f**: specific leaf area (SLA) under control and drought conditions, respectively. Data are mean ± SE. Data labeled with different letters are significantly different at *P* ≤ 0.05

Under control condition, leaf fresh weight (FW) was significantly reduced at ML and LL compared to that at FL in *A. halimus*. Leaf FW was significantly higher at ML and LL than that at FL in *A. nummularia* (the maximum FW was at ML). In *A. portulacoides* and *A. prostrata*, leaf FW was significantly higher at ML than that at FL and LL, which were significantly similar (Fig. 1a). Under drought condition, leaf FW increased significantly at ML and LL compared to that at FL in the four species (Fig. 1b).

Under control condition, leaf dry weight (DW) was significantly higher at FL than that at ML and LL in *A. halimus*. In *A. nummularia*, the maximum leaf DW was detected at ML compared to that at FL and LL. Leaf DW deceased significantly at ML and LL compared to that at FL in *A. portulacoides* and *A. prostrata* (Fig. 1c). Under drought condition, leaf DW was significantly higher at ML and LL than that at FL in *A. halimus* and *A. nummularia*. In contrast, leaf DW was significantly lower at ML and LL than that at FL in *A. portulacoides* and *A. prostrata* (Fig. 1d).

Under control condition, SLA increased significantly at ML and LL compared to that at FL in all species (Fig. 1e). Under drought condition, SLA was significantly higher at FL than that at LL and ML, respectively in *A. halimus*. SLA was significantly higher at ML and LL than that at FL in *A. nummularia*, *A. portulacoides* and *A. prostrata* (Fig. 1f).

### Diurnal regulation of photosynthetic rate (*A*) and stomatal conductance (*g*_*s*_) in response to different light intensities under control and drought conditions

**Fig. 2:**
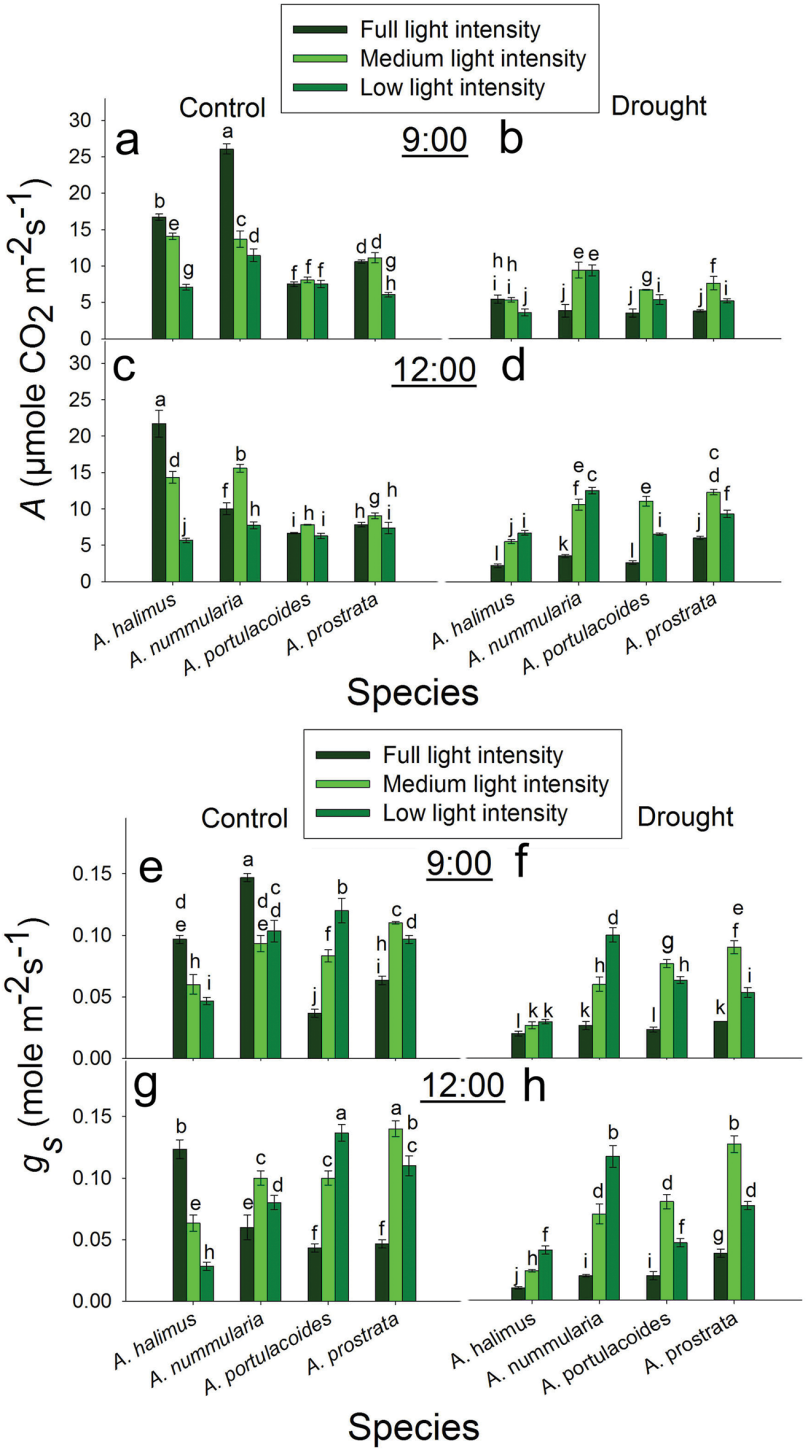
Diurnal effect of different light intensities on photosynthetic rate (*A*) and stomatal conductance (*g*_*s*_) under control and drought conditions. **a** and **b**: photosynthetic rate (*A*) under control and drought conditions, respectively at 9:00. **c** and **d**: photosynthetic rate (*A*) under control and drought conditions, respectively at 12:00. **e** and **f**: stomatal conductance (*g*_*s*_) under control and drought conditions, respectively at 9:00. **g** and **h**: stomatal conductance (*g*_*s*_) under control and drought conditions, respectively at 12:00. Data are mean ± SE. Data labeled with different letters are significantly different at *P* ≤ 0.05

At 9:00 under control condition, *A* decreased significantly and progressively at ML and LL compared to that at FL in *A. halimus* and *A. nummularia*. In *A. portulacoides*, there was no significant change in *A* at all light intensities. In *A. prostrata*, *A* decreased significantly at LL compared to that at FL and ML (which recorded a significantly, similar value of *A*, Fig. 2a). At 9:00 under drought condition, rate of *A* was significantly similar at FL and ML and higher than that at LL in *A. halimus*. *A* was significantly lower at FL than that at ML and LL in *A. nummularia*, *A. portulacoides* and *A. prostrata* (Fig. 2b).

At 12:00 under control condition, *A* declined significantly and progressively with the decrease in the light intensity in *A. halimus*. Rate of *A* peaked significantly at ML compared to FL and LL in *A. nummularia*, *A. portulacoides* and *A. prostrata* (Fig. 2c). At 12:00 under drought condition, the rate of *A* was significantly higher at ML and LL than that at FL in all species (Fig. 2d).

At 9:00 under control condition, *g*_*s*_ decreased significantly and progressively with the decrease in the light intensity in *A. halimus*. In *A. nummularia*, *g*_*s*_ peaked significantly at FL compared to that at ML and LL. Stomatal conductance (*g*_*s*_) increased significantly at ML and LL compared to FL in *A. portulacoides* and *A. prostrata* (Fig. 2e). At 9:00 under drought condition, *g*_*s*_ increased significantly at ML and LL compared to that at FL in the *Atriplex* species (Fig. 2f).

At 12:00 under control condition, *g*_*s*_ decreased significantly and progressively with the decrease in the light intensity in *A. halimus*. Stomatal conductance (*g*_*s*_) was significantly higher at ML and LL than that at FL in *A. nummularia*, *A. portulacoides* and *A. prostrata* (Fig. 2g). At 12:00 under drought condition, *g*_*s*_ increased significantly at ML and LL compared to that at FL in the four species (Fig. 2h).

### Diurnal effect of different light intensities on leaf pigments under control and drought conditions

**Fig. 3:**
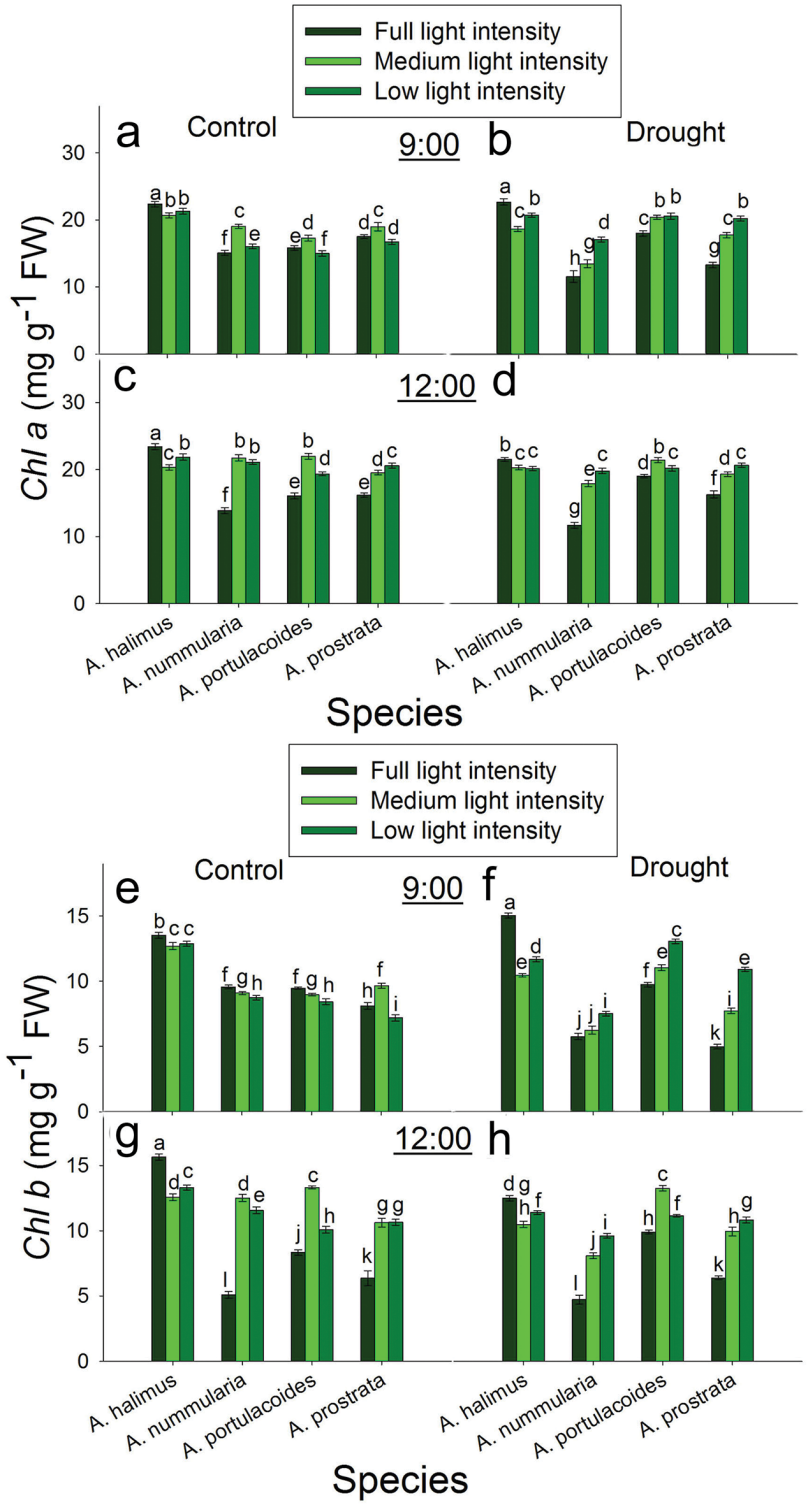
Diurnal effect of different light intensities on chlorophyll a (*Chl a*) and chlorophyll b (*Chl b*) under control and drought conditions. **a** and **b**: chlorophyll a under control and drought conditions, respectively at 9:00. **c** and **d**: chlorophyll a under control and drought conditions, respectively at 12:00. **e** and **f**: chlorophyll b under control and drought conditions, respectively at 9:00. **g** and **h**: chlorophyll b under control and drought conditions, respectively at 12:00. Data are mean ± SE. Data labeled with different letters are significantly different at *P* ≤ 0.05

At 9:00 under control condition, chlorophyll a (*Chl a*) decreased significantly at ML and LL compared to that at FL in *A. halimus*. Chlorophyll a peaked significantly at ML compared to those at FL and LL in *A. nummularia*, *A. portulacoides* and *A. prostrata* (Fig. 3a). At 9:00 under drought condition, *Chl a* was significantly higher at FL than that at ML and LL in *A. halimus*. Chlorophyll a increased significantly and progressively with the decrease in the light intensity in *A. nummularia* and *A. prostrata*. Chlorophyll a was significantly similar at ML and LL, which was higher than that at FL in *A. portulacoides* (Fig. 3b). At 12:00 under control and drought conditions, *Chl a* decreased significantly at ML and LL compared to that at FL in *A. halimus*. Chlorophyll a was significantly higher at ML and LL than that at FL in *A. nummularia*, *A. portulacoides* and *A. prostrata* (Fig. 3c, d).

At 9:00 under control condition, chlorophyll b (*Chl b*) was significantly reduced at ML and LL compared to that at FL in *A. halimus*, *A. nummularia* and *A. portulacoides*. Chlorophyll b peaked significantly at ML compared to those at FL and LL in *A. prostrata* (Fig. 3e). At 9:00 under drought condition, *Chl b* decreased significantly at ML and LL compared to that at FL in *A. halimus*. In *A. nummularia*, *Chl b* was significantly similar at FL and ML, which was lower than that at LL. Chlorophyll b increased significantly and progressively with the decrease in the light intensity in *A. portulacoides* and *A. prostrata* (Fig. 3f). At 12:00 under control and drought conditions, *Chl b* decreased significantly at ML and LL compared to that at FL in *A. halimus*. Chlorophyll b increased significantly at ML and LL compared to that at FL in *A. nummularia*, *A. portulacoides* and *A. prostrata* (Fig. 3g, h).

### Diurnal effect of different light intensities on total soluble sugar (TSS) and starch under control and drought conditions

**Fig. 4:**
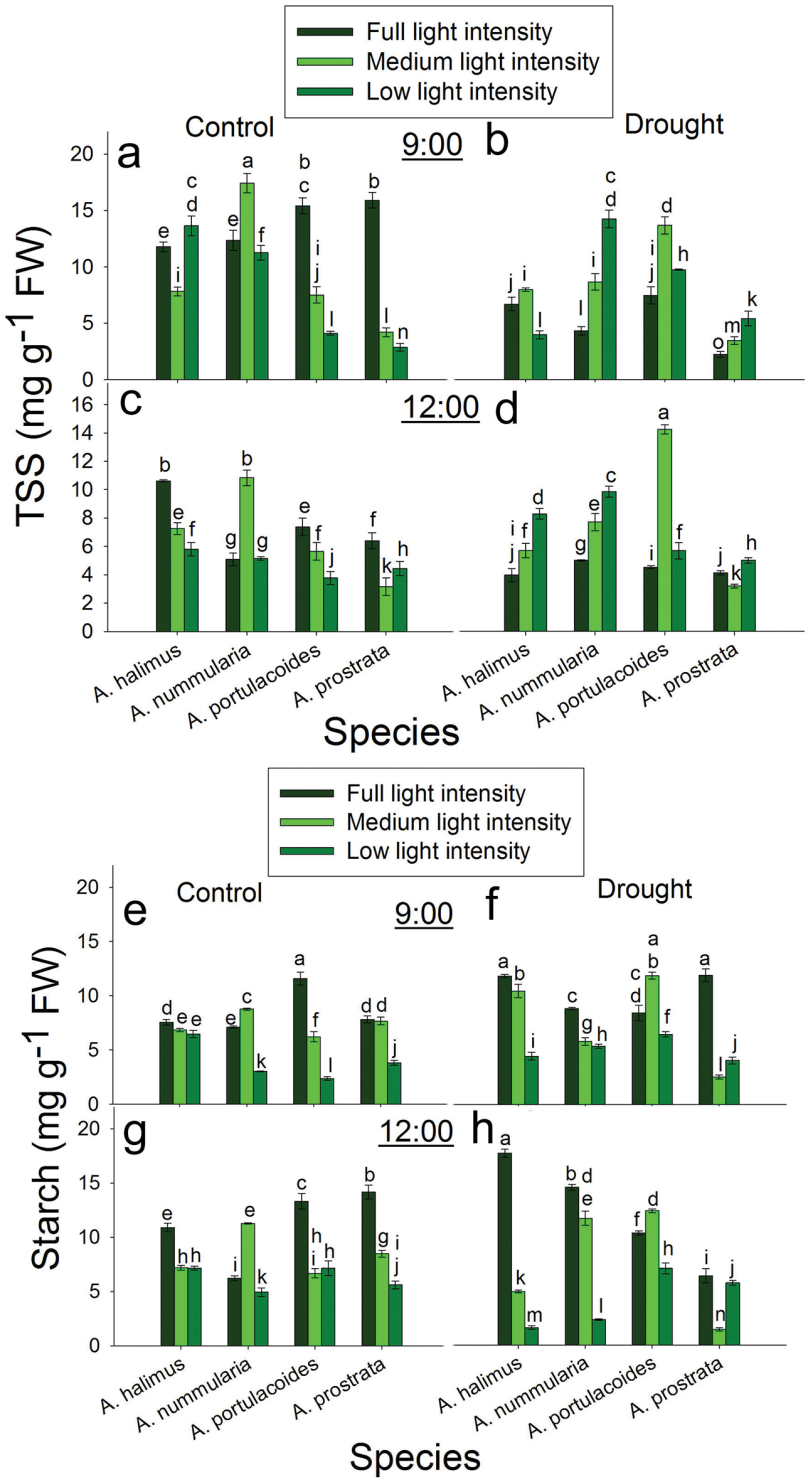
Diurnal effect of different light intensities on total soluble sugar (TSS) and starch content under control and drought conditions. **a** and **b**: TSS content under control and drought conditions, respectively at 9:00. **c** and **d**: TSS content under control and drought conditions, respectively at 12:00. **e** and **f**: starch content under control and drought conditions, respectively at 9:00. **g** and **h**: starch content under control and drought conditions, respectively at 12:00. Data are mean ± SE. Data labeled with different letters are significantly different at *P* ≤ 0.05

At 9:00 under control condition, TSS was significantly higher at LL than that at FL and ML in *A. halimus*. TSS peaked significantly at ML compared to that at FL and LL in *A. nummularia*. TSS was significantly lower at ML and LL than that at FL in *A. portulacoides* and *A. prostrata* (Fig. 4a). At 9:00 under drought condition, TSS was significantly lower at LL than that at FL and ML in *A. halimus*. TSS was significantly higher at ML and LL than that at FL in *A. nummularia*, *A. portulacoides* and *A. prostrata* (Fig. 4b).

At 12:00 under control condition, TSS was significantly lower at ML and LL than that at FL in *A. halimus*, *A. portulacoides* and *A. prostrata.* TSS peaked significantly at ML compared to that at FL and LL in *A. nummularia* (Fig. 4c). At 12:00 under drought condition, TSS was significantly higher at ML and LL than that at FL in the four species except *A. prostrata* in which, TSS was significantly reduced at ML compared to that at FL and LL (Fig. 4d).

At 9:00 under control condition, starch content was significantly lower at ML and LL than that at FL in *A. halimus* and *A. portulacoides.* Starch content peaked significantly at ML in *A. nummularia* compared to FL and LL. Starch content was significantly similar at FL and LL, which was significantly higher than that at LL in *A. prostrata* (Fig. 4e). At 9:00 under drought condition, starch content was significantly lower at ML and LL than that at FL in *A. halimus*, *A. nummularia* and *A. prostrata*. Starch content peaked significantly at ML compared to that at FL and LL in *A. portulacoides* (Fig. 4f).

At 12:00 under control condition, starch content was significantly lower at ML and LL than that at FL in *A. halimus*, *A. portulacoides* and *A. prostrata.* Starch content peaked significantly at ML compared to that at FL and LL in *A. nummularia* (Fig. 4g). At 12:00 under drought condition, starch content followed the same trend like that at 9:00 under drought condition (Fig. 4h).

### Diurnal effect of different light intensities on Rubisco protein content under control and drought conditions

**Fig. 5:**
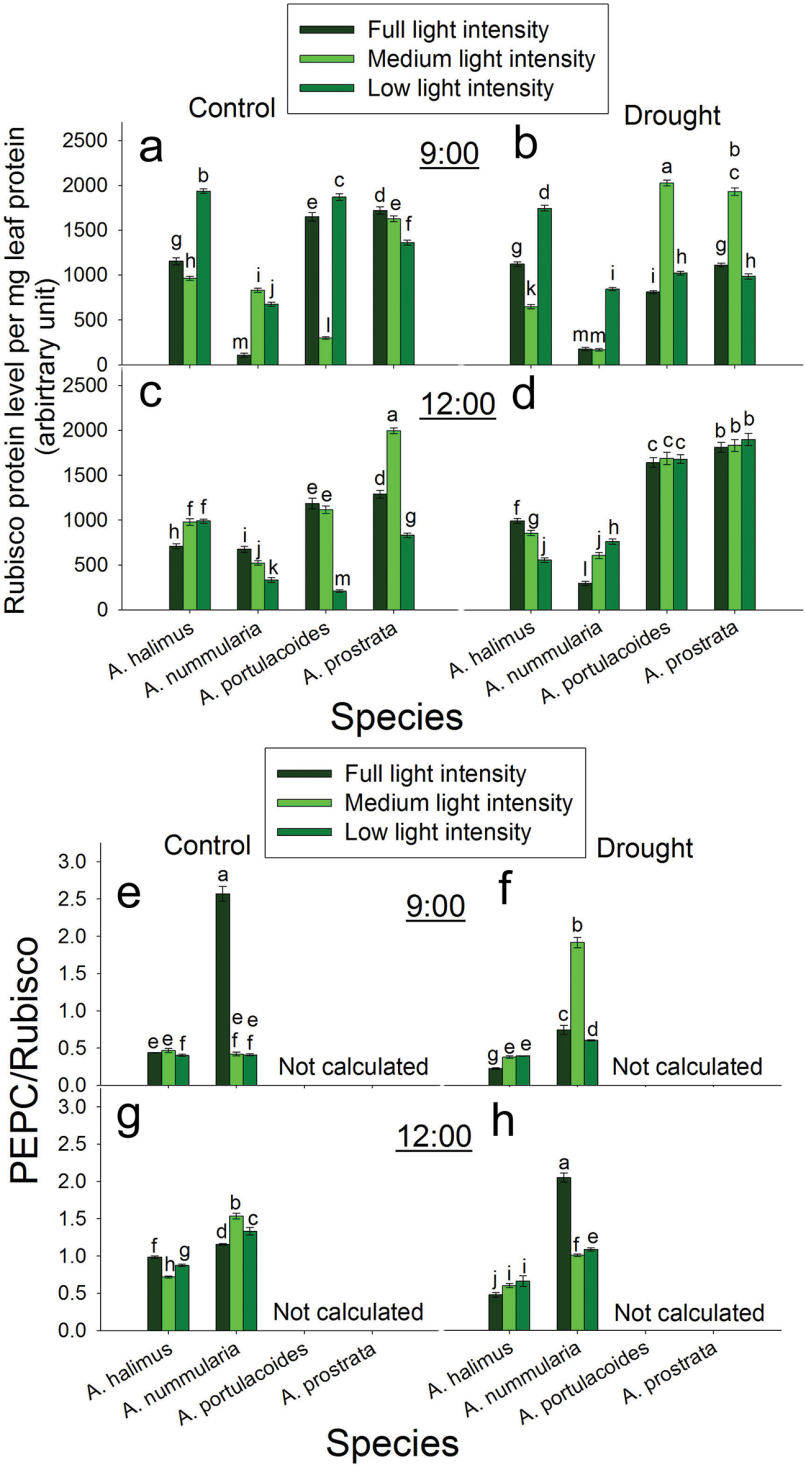
Diurnal effect of different light intensities on Rubisco protein level and PEPC/ Rubisco ratio under control and drought conditions. **a** and **b**: Rubisco protein content under control and drought conditions, respectively at 9:00. **c** and **d**: Rubisco protein content under control and drought conditions, respectively at 12:00. **e** and **f**: PEPC/ Rubisco ratio under control and drought conditions, respectively at 9:00. **g** and **h**: PEPC/ Rubisco ratio under control and drought conditions, respectively at 12:00. Data are mean ± SE. Data labeled with different letters are significantly different at *P* ≤ 0.05

At 9:00 under control condition, Rubisco protein content was significantly higher at LL than that at FL and ML in *A. halimus* and *A. portulacoides*. Rubisco protein content was significantly higher at ML and LL than that at FL in *A. nummularia*. In *A. prostrata*, Rubisco protein content decreased significantly and progressively with the decrease in the light intensity (Fig. 5a). At 9:00 under drought condition, Rubisco protein content was significantly higher at LL than that at FL and ML in *A. halimus* and *A. nummularia*. Rubisco protein content peaked significantly at ML compared to that at FL and LL in *A. portulacoides* and *A. prostrata* (Fig. 5b).

At 12:00 under control condition, Rubisco protein content increased significantly at ML and LL compared to that at FL in *A. halimus*. Rubisco protein content decreased significantly and progressively with the decrease in the light intensity in *A. nummularia*. Rubisco protein level was significantly lower at LL than that at FL and ML in *A. portulacoides*. Rubisco protein content peaked significantly at ML compared to that at FL and LL in *A. prostrata* (Fig. 5c). At 12:00 under drought condition, Rubisco protein content decreased significantly and progressively with the decrease in the light intensity in *A. halimus*. In contrast, Rubsico protein content increased significantly and progressively with the decrease in the light intensity in *A. nummularia*. There was no significant change in Rubisco protein content at the different light intensities in *A. portulacoides* and *A. prostrata* (Fig. 5d).

### Diurnal effect of different light intensities on PEPC/ Rubisco ratio under control and drought conditions

At 9:00 under control condition, PEPC/ Rubisco ratio was significantly lower at LL than that at FL and ML in *A. halimus*. PEPC/ Rubisco ratio peaked significantly at FL compared to that at ML and LL in *A. nummularia* (Fig. 5e). At 9:00 under drought condition, PEPC/ Rubisco ratio was significantly lower at FL than that at ML and LL in *A. halimus*. PEPC/ Rubisco ratio peaked significantly at ML compared to that at FL and LL (Fig. 5f).

At 12:00 under control condition, PEPC/ Rubisco ratio was significantly higher at FL than that at ML and LL in *A. halimus*. PEPC/ Rubisco ratio peaked significantly at ML compared to that at FL and LL in *A. nummularia* (Fig. 5g). At 12:00 under drought condition, PEPC/ Rubisco ratio was significantly higher at ML and LL than that at FL in *A. halimus*. PEPC/ Rubisco peaked significantly at FL compared to ML and LL in *A. nummularia* (Fig. 5h).

### Effect of different light intensities on number of bundle sheath cells with chloroplasts (BSC) under control and drought conditions

**Fig. 6:**
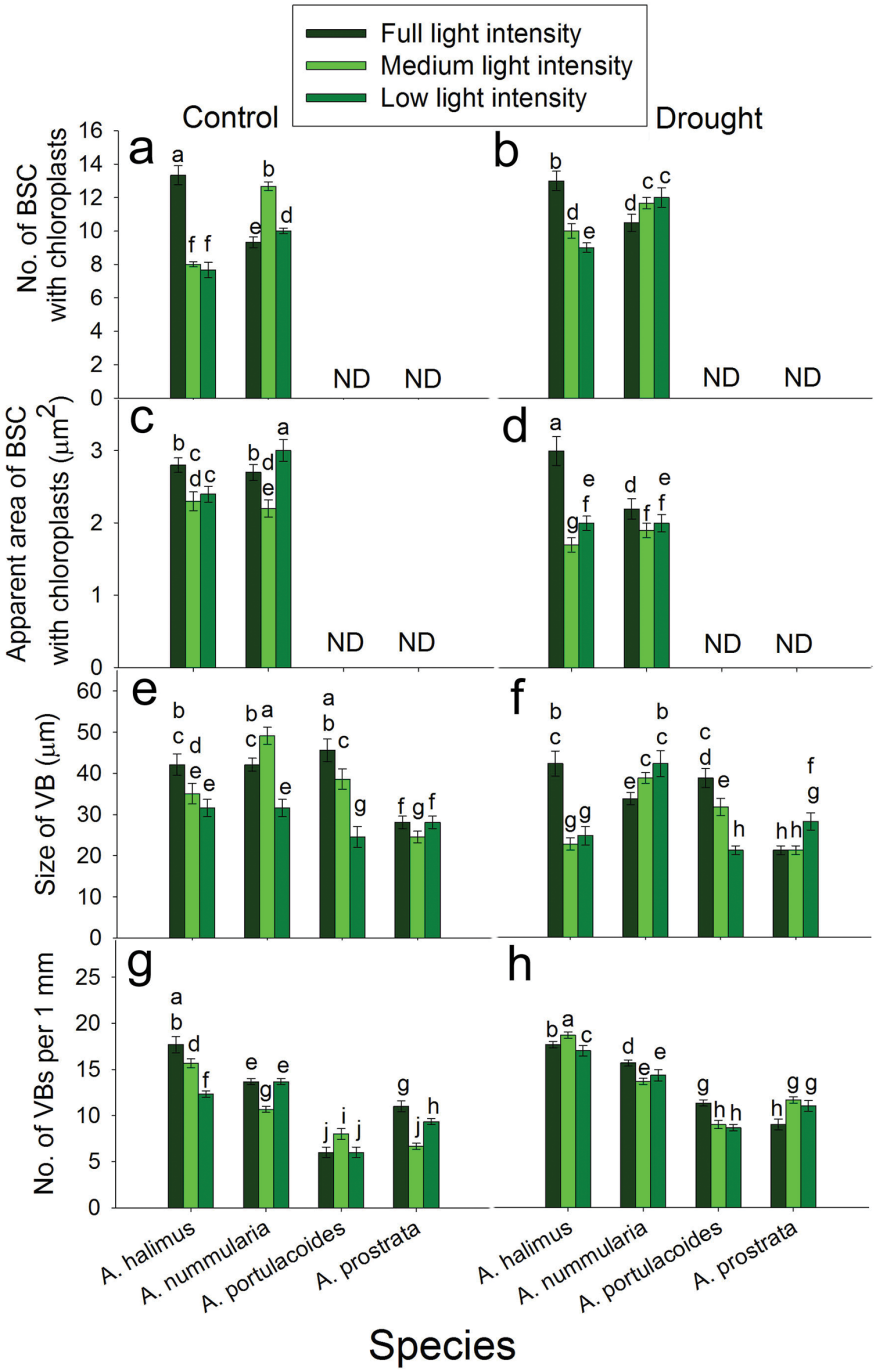
Effect of different light intensities on leaf anatomical features. **a** and **b**: number of bundle sheath cells (BSC) with chloroplast under control and drought conditions, respectively. **c** and **d**: apparent area of bundle sheath cells with chloroplasts (BSC) under control and drought conditions, respectively. **e** and **f**: size of vascular bundle (VB) under control and drought conditions, respectively. **g** and **h**: number of vascular bundles (VBs) under control and drought conditions, respectively. Data are mean ± SE. Data labeled with different letters are significantly different at *P* ≤ 0.05

Under control condition, the number of BSC was significantly higher at FL than that at ML and LL in *A. halimus*. Number of BSC peaked significantly at ML compared to that at FL and LL in *A. nummularia* (Fig. 6a). Under drought condition, number of BSC decreased significantly and progressively with the decrease in the light intensity in *A. halimus*. Number of BSC increased significantly at ML and LL compared to that at FL in *A. nummularia* (Fig. 6b).

### Effect of different light intensities on the number of apparent areas of bundle sheath cells with chloroplasts (BSC) under control and drought conditions

Under control condition, apparent area of BSC was significantly higher at FL than that at ML and LL in *A. halimus*. The apparent area of BSC peaked significantly at LL compared to that at FL and ML in *A. nummularia* (Fig. 6c). Under drought condition, the apparent area of BSC was significantly lower at ML and LL than that at FL in *A. halimus* and *A. nummularia* (Fig. 6d).

### Effect of different light intensities on size of vascular bundle (VB) under control and drought conditions

Under control condition, size of VB was significantly higher at FL than that at ML and LL in *A. halimus* and *A. portulacoides*. Size of VB peaked significantly at ML compared to that at FL and LL in *A. nummularia*. Size of VB was significantly reduced at ML compared to that at FL and LL in *A. prostrata* (Fig. 6e). Under drought condition, the size of VB was significantly higher at FL than that at ML and LL in *A. halimus* and *A. portulacoides*. The size of VB was lower at FL than that at ML and LL in *A. nummularia*. The size of VB was significantly higher at LL than that at FL and ML in *A. prostrata* (Fig. 6f).

### Effect of different light intensities on the number of vascular bundles (VBs) under control and drought conditions

Under control condition, the number of VBs decreased significantly and progressively with the decrease in the light intensity in *A. halimus*. Number of VBs was significantly lower at ML than that at FL and LL in *A. nummularia* and *A. prostrata*. Number of VBs peaked significantly at ML compared to that at FL and LL in *A. portulacoides* (Fig. 6g). Under drought condition, number of VBs was significantly higher at ML, FL and LL, respectively in *A. halimus*. Number of VBs was significantly higher at FL than that at ML and LL in *A. nummularia* and *A. portulacoides*. Number of VBs was significantly higher at ML and LL than that at FL in *A. prostrata* (Fig. 6h).

### Diurnal regulation of the transcript level of alternative oxidase (*AOX*) and ferrodoxin (NADPH/NADP) reductase (*FNR*) at different light intensities under control and drought conditions

**Fig. 7:**
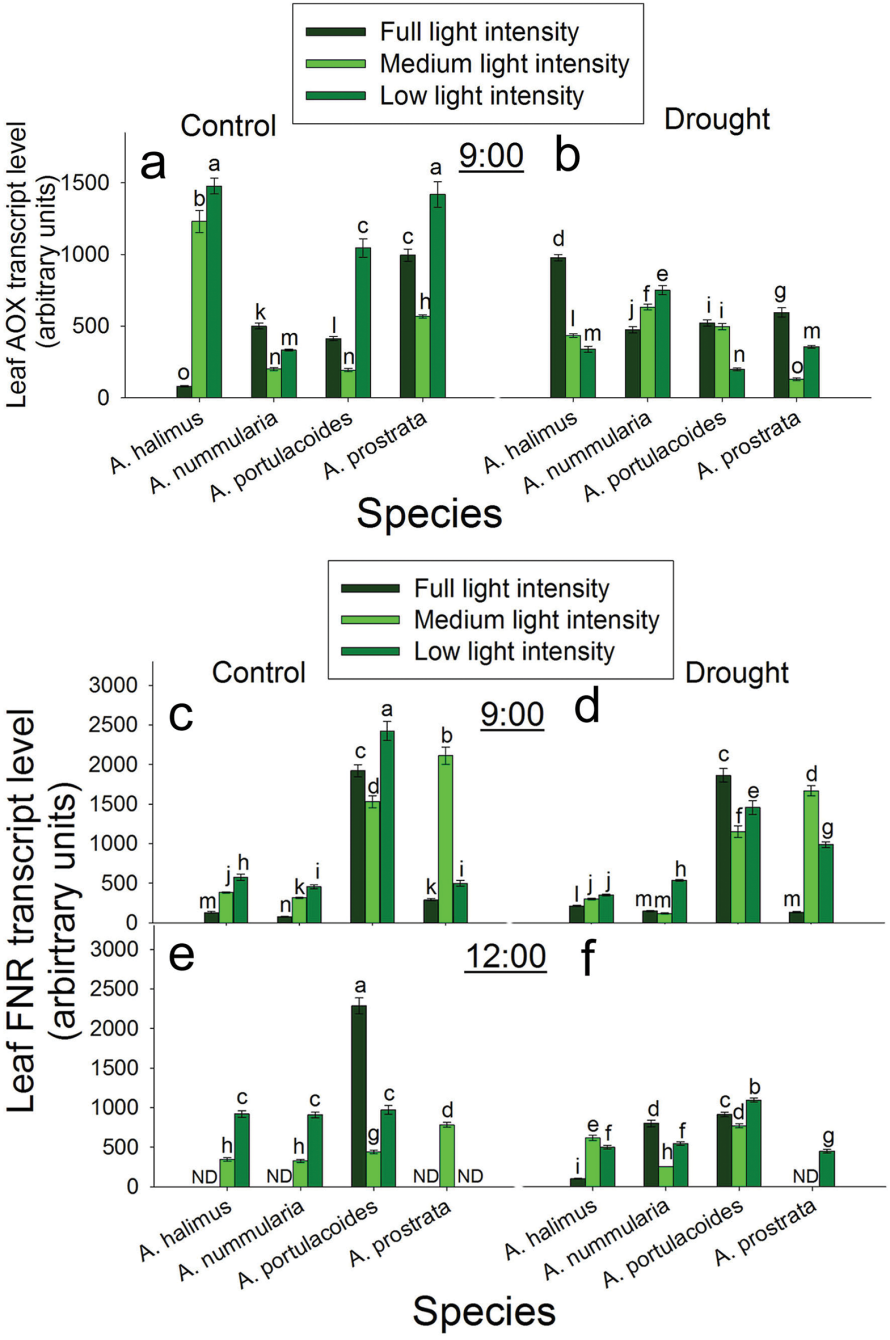
Diurnal effect of different light intensities on leaf *AOX* and *FNR* transcript level under control and drought conditions. **a** and **b**: *AOX* transcript level under control and drought conditions, respectively at 9:00. **c** and **d**: *FNR* transcript level under control and drought conditions, respectively at 9:00. **e** and **f**: *FNR* transcript level under control and drought conditions, respectively at 12:00. Data are mean ± SE. Data labeled with different letters are significantly different at *P* ≤ 0.05

At 9:00 under control condition, the transcript level of *AOX* increased highly and significantly at ML and LL compared to that at FL in *A. halimus*. The transcript level of *AOX* was significantly down-regulated at ML and LL compared to that at FL in *A. nummularia*. The transcript level of *AOX* increased significantly and gradually at LL, FL and LL, respectively in *A. portulacoides* and *A. prostrata* (Fig. 7a). At 9:00 under drought condition, the transcript level of *AOX* was significantly down-regulated at ML and LL compared to that at FL in *A. halimus*. The transcript level of *AOX* was significantly up-regulated at ML and LL compared to that at FL in *A. nummularia*. The transcript level of *AOX* was significantly down-regulated at LL only compared to that at FL and ML in *A. portulacoides*. The transcript level of *AOX* was significantly higher at FL than that at ML and LL in *A. prostrata* (Fig. 7b).

At 12:00, the transcript level of *AOX* was undetected in the four species under control and drought conditions.

At 9:00 under control condition, the transcript level of *FNR* increased significantly and progressively with the decrease in the light intensity in *A. halimus* and *A. nummularia*. The transcript level of *FNR* was significantly higher at LL and FL than that at ML in *A. portulacoides*. The transcript level of *FNR* peaked significantly at ML in *A. prostrata* (Fig. 7c). At 9:00 under control condition, the transcript level of *FNR* was significantly down-regulated at FL compared to that at ML and LL in *A. halimus*. The transcript level of *FNR* was significantly down-regulated at FL and ML compared to that at LL in *A. nummularia*. Similar trend as that under control condition was recorded for *A. portulacoides* and *A. prostrata* (Fig. 7d).

At 12:00 under control condition, the transcript level of *FNR* was not detected at FL and it was significantly higher at LL than that at ML in *A. halimus* and *A. nummularia*. The transcript level of *FNR* was significantly and highly up-regulated at FL compared to that at ML (the lowest level) and LL in *A. portulacoides*. The transcript level of *FNR* was only detected at ML in *A. prostrata* (Fig. 7e). At 12:00 under drought condition, the transcript level of *FNR* was significantly up-regulated at ML and LL compared to that at FL in *A. halimus*. The transcript level of *FNR* was significantly up-regulated at FL compared to that at ML and LL in *A. nummularia*. The transcript level of *FNR* was significantly higher at LL than that at FL and ML (the lowest level) in *A. portulacoides*. The transcript level of *FNR* was only detected at LL in *A. prostrata* (Fig. 7f).

## Discussion

### Response of the C4 and C3 species to different light intensities under control condition

C4 plants have a higher photosynthetic rate compared to their C3 counterparts under conditions of high light intensities and high temperature, but C3 plants have advantages over C4 plants under low light intensity, high carbon dioxide levels and low temperature (e.g. Pearcy and Ehleringer 1984). However, it has found contrasting results for the response and efficiency of the photosynthesis in the C4 plants under low light intensity (see introduction). In the present study, the photosynthetic rate (*A*) in *A. halimus* and *A. nummularia* was positively correlated with *g*_*s*_. However, *A* significantly peaked at FL at 12:00 and at 9:00 in *A. halimus* and *A. nummularia*, respectively, implying that *A. halimus* leaves follow an anisohydric behaviour but the leaves of *A. nummularia* follow isohydric strategy. Stomatal conductance (*g*_*s*_) was positively correlated to light intensity under control condition in *A. halimus* (Nada et al. 2018).

It was hypothesized that the acclimation of photosynthesis to low light intensity in C4 NAD-ME spp. manifested as a greater down-regulation of photosynthetic rate and higher leakiness (Sonawane et al. 2018). *Atriplex halimus* at ML could be considered as a typical C4 NAD-ME sp. in which the acclimation to ML involves a reduction in *A* and keeping it in a stable trend at 9:00 and 12:00. Meanwhile, at LL, *A. halimus* followed isohydric behavior and limited the *g*_*s*_ despite the increase in light intensity at 12:00. However, in *A. nummularia* at FL, *g*_*s*_ could be negatively affected by the increase in the natural light intensity (at noon) meanwhile, this noon light positively enhanced *g*_*s*_ and hence *A* at ML (Fig. 2); suggesting *g*_*s*_ and *A* were positively regulated by moderate light intensity at 9:00 (FL) or at 12:00 (ML) in *A. nummularia*, which contrasts with *A. halimus*.

Sugars provide energy for plant growth, metabolism and reproduction, and also they play vital role in regulating plant adaptation to environmental changes. Changes in light intensities affect the metabolism of sugars, which consequently affects the accumulation of sugars in plants (Wu et al. 2025). Sugars act as signalling molecules by interacting with other molecules to regulate plants growth and development and the whole physiological processes of plants (Mukarram et al. 2023). The accumulation of TSS (Fig. 4) at 9:00 at LL in *A. halimus* could be a result of the increase in *A* but this could trigger the decrease in *A* at 12:00. The accumulation of the end product of photosynthesis could negatively affect *A* (Cheng et al. 2009). However, starch at LL was not accumulated at 9:00 and 12:00 in *A. halimus*. Conversion of the photosynthetic assimilates into starch at low light intensity seems to be species dependent because of the presence of many contradictions in this context (Tang et al. 2022; Proietti et al. 2023). At low light intensity, accumulation of soluble sugars instead of starch could be an acclimation strategy for producing enough ATP. The increase in relative water content (data not shown) in the C4 spp. at LL may suggest that the decrease in *g*_*s*_ was not due to lower leaf water content, but perhaps because of the increase in *C*_*i*_ (Engineer et al. 2016) and to reduce the effect of photorespiration resulting from the exposure to low light intensity. The regulation of *g*_*s*_ could be affected by PEPC/ Rubisco ratio and the activity of PEPC (Cousins et al. 2007). The accumulation pattern of sugars was not consistent for all studied species under control and drought conditions at different light intensities, suggesting that this pattern could be dependent on the species.

The transcript level of *AOX* (Fig. 7) was up-regulated at ML and LL at 9:00 with a higher level at LL in *A. halimus*. Meanwhile, *AOX* transcript level was reduced at ML and LL in *A. nummularia*. It was suggested that total soluble sugar and other factors like growth light intensity could be directly related with *AOX* activity (Florez-Sarasa et al. 2009). However, this was not consistent with the present data, where the regulation of *AOX* was related to TSS in *A. halimus* at LL only, suggesting other factors could affect the regulation of *AOX*. The up-regulation of *AOX* may suggest oxidative stress. Low light intensity could produce oxidative stress and increase the activity of antioxidants (Zhu et al. 2017).

The exposure of C4 plants to low irradiance could cause an increase in photorespiration due to the decrease in CO_2_ in the bundle sheath (CO_2_ leakage). However, smaller bundle sheath cells and rearrangement of chloroplasts in bundle sheath cells and mesophyll cells could potentially lead to effective light absorption and potential acclimation to low light intensity in C4 species (Bellasio and Ermakova 2022). Therefore, CO_2_ leakage could be one of the main factors that control *A* in these C4 species especially at LL. It is expected that *A. halimus* at ML and LL could suffer from oxidative stress. However, this was not the case of *A. nummularia* where ML enhanced *A* and *g*_*s*_ at 12:00. However, a large number with a small size of BSC (Fig. 6) and chloroplast rearrangement at ML in *A. nummlaria* could explain the enhancement of *A.* Meanwhile, the decrease in number and increase in size of BSC at LL, suggest that *A. nummularia* could cope with CO_2_ leakage at ML but not at LL. A smaller bundle sheath enhanced the assimilation rate at low light intensity to the level of that at high light intensity in *Setaria viridis* (NADP-ME) (Bellasio and Ermakova 2022).

Optimization of PEPC/ Rubisco ratio was suggested to be an effective factor in minimizing the CO_2_ leakiness from BS and enhancing the CO_2_ assimilation rate (Kromdijk et al. 2008). In *A. nummularia*, the increase in PEPC/ Rubisco (Fig. 5) ratio has coincided with the increase in *A* at FL at 9:00 and ML at 12:00. However, the increase in this ratio at LL at 12:00 did not positively affect *A*, perhaps due to the non-adjusted bundle sheath cells and CO_2_ leakiness. The decrease in this ratio at ML and LL at 12:00 in *A. halimus* could be another reason for reducing *A* compared to that at FL (Engineer et al. 2016 and ref. therein).

One of the most common strategies used by most plants to acclimate to low light intensities is increasing the levels of *Chl a* and *Chl b* to increase the light-capturing capacities (Tang et al. 2022). *A. nummularia* showed an increase in *Chl a* and *Chl b* (Fig. 3) at ML and LL compared to FL, especially at 12:00. However, *A. halimus* did not follow this strategy.

The photosynthetic rate (*A*) (Fig. 2) and the photosynthetic efficiency (Table S1) were significantly lower in the C3 than the C4 species at comparable light intensities. In contrast to the C4 species, *A* was not positively correlated to *g*_*s*_ in the C3 species, where the increase in *g*_*s*_ at noon did not reflect on the increase in *A* at all light intensities. Moreover, *g*_*s*_ highly and significantly increased at ML and LL at 9:00 and 12:00, but there was no significant increase in *A* in *A. portulacoides* and even there was a reduction in *A* at LL in *A. prostrata* at 9:00. At 12:00, the increase in *A* at ML was about 16–28% of that at FL, although there was an increase of about two and a half to threefold in *g*_*s*_ in both species. These unparalleled increases led to a significant accumulation of *C*_*i*_ (Fig. S7). These data suggest that *g*_*s*_ was negatively affected by light intensity at noon at FL but at ML and LL, the enhanced *g*_*s*_ could be attributed to high leaf water content (data not shown) with no effect of the low light intensity. In contrast to our data, most of the previous studies found a decrease in *A* and *g*_*s*_ at low light intensities, although there was an increase in leaf water potential (Almeida et al. 2020; Tang et al. 2022; Proietti et al. 2023). Zhu et al. (2017) found an increase in *g*_*s*_ and *C*_*i*_ after five days from exposing *Brassica camperstris* to low light compared to that at full light, although, there was no significant increase in *A* and they attributed that to an inefficient electron transport system. Also, they found a reduction in *g*_*s*_ and *C*_*i*_ after ten days from applying low light intensity. It could be said that *A* was limited by other internal factor rather than *g*_*s*_. Moreover, low light intensity may uncouple the relationship between *g*_*s*_ and *C*_*i*_. Opening of stomata could be regulated by different pathways, including one pathway of internal CO_2_ under red light (the relationship between stomatal conductance and internal CO_2_ was reviewed in Engineer et al. 2016).There was an increase in *Chl a* and *Chl b* at 12:00, but there was no increase in *A*. Also, there was no consistent relationship between Rubisco content and *A* at 9:00 and 12:00, but the photosynthetic efficiency was higher at ML. In contrast to the C4 species in the present study, the decrease in TSS at ML and LL at 9:00 did not enhance *A* at 12:00 but there was another decrease in TSS as a result of the decline in *A*. However, the enhanced *A* at ML compared to that at FL and LL at 12:00 could be attributed to the increase in *Chl a* and *Chl b* and Rubisco protein content in both C3 species. As mentioned before, low light intensity could initiate oxidative stress; the transcript level of *AOX* at ML was lower. The increase in *A* was not mirrored in leaf dry weight (Fig. 1), where the increase in fresh weight was due to higher water content not producing dry biomass.

### Response of the C4 and C3 species to different light intensities under drought condition

Under drought condition, *A* and *g*_*s*_ (Fig. 2) were significantly reduced compared to that under control condition at all light intensities except for that at LL at 12:00 for *A. halimus* and *A. nummularia*. At 12:00, the lowest reduction in *g*_*s*_ was detected at FL, suggesting that *g*_*s*_ was mainly affected by soil water content (Nada et al. 2018). However, under ML and LL, *g*_*s*_ significantly increased, which could be attributed to the decline of evapotranspiration (Esmaili et al. 2020) and saving the soil water content more than that at FL. This increase in *g*_*s*_ has coincided with another increase in *A*. It seems that LL was better for *A. halimus* under drought condition, where leaf FW and DW (Fig. 1) were significantly the highest. It was suggested that SLA declined with the increase in light intensities (Ghorbanzadeh et al. 2021). However, in *A. halimus*, SLA increased at FL; perhaps it was an attempt to increase the area for stomata, but the final result was lower leaf content (data not shown), and lower *g*_*s*_ and *A*. Compared to that under the control condition, the number of BSC increased, but the apparent area of BSC decreased at ML and LL. Therefore, enhanced *A* could be attributed to the smaller BSC compared to that at FL under drought condition but this was not the case under control condition. The small size of BSC at ML and LL compared to that at FL under control condition did not enhance the assimilation rate, suggesting that the increase in accumulation of internal CO_2_ due to higher *g*_*s*_ (compared to that at drought) and BSC leakage could negatively affect the photosynthetic apparatus. Therefore, improving *A* may be due to a balanced reduction in *g*_*s*_ and internal CO_2_ in addition to the lower size of BSC and optimized PEPC/ Rubisco ratio that could cope with the negative effect of CO_2_ leakiness and positively affect the assimilation rate compared to that at FL under drought condition. The downregulation of *AOX* at ML and LL could support this conclusion.

Again, it seems that *Chl a* and *Chl b* (Fig. 3) did not play a role in *A. halimus* acclimation to lower light intensities. Similar to that under control condition, *A* and *g*_*s*_ showed no change at 9:00 and 12:00 at ML. Meanwhile, *A* and *g*_*s*_ were enhanced at 12:00 at LL, which contrasts with that under the control condition. The decrease in TSS at 9:00 could positively trigger *A* at noon. Therefore, drought stress could be alleviated by the exposure to a lower irradiance in *A. halimus*. For *A. nummularia*, optimized ratio of *Chl a* and *Chl b*, increased the number of bundle sheath cells and decreased the area of them, whcih could be factors involved in enhancing the assimilation rate *A* at ML and LL under drought condition. However, PEPC/ Rubisco ratio did not show any increase at ML and LL compared to FL. Sonawane et al. (2018) found a reduction in the assimilation rate in NADP-ME, NAD-ME and PEPCk species, considering this reduction was an acclimation to shade. These species acclimated to shade by reducing Rubisco and PEPC protein content and activity. Moreover, it was suggested that reduced Rubisco protein content is a common photosynthetic acclimation to shade to allow optimal nitrogen allocation for maximal light harvesting (Evans and Poorter 2001; Walters 2005). However, in the present study, there was no consistent relationship between *A* and the change in Rubisco and PEPC protein contents at ML or LL in *A. halimus* and *A. nummularia* under control and drought conditions, suggesting two conclusions: first, the ratio of PEPC/ Rubisco should be optimized for efficient assimilation rate and second, there is no need to reduce PEPC and Rubisco for better acclimation to shade where low light intensities (ML and LL) could be appropriate for the growth of these C4 NAD-ME species under drought condition and for *A. nummularia* at 12:00 under control condition.

The increase in TSS (ATP production, see above) in these C4 species could lead to enhanced adaptation performance of these species under drought condition. In contrast, the accumulation of starch as the end product of photosynthesis as a result of growth inhibition could limit the photosynthetic rate. A lower growth rate at FL could suggest lower sink demand, leading to starch accumulation in the source leaf (Cheng et al. 2009 and ref. therein). However, the source-sink relationship could be modified according to changes in the photoperiod. Reducing the light period decreased the end product in the source leaf, which was similar to increasing sink demand (Li et al. 2007), leading to enhanced *A* at ML and LL compared to FL, especially at 12:00 under drought condition.

Another acclimation factor to drought stress is reducing SLA. High SLA means thinner leaf and wider leaf; exposure to low light intensity leads to increasing SLA for increasing the efficiency of light capturing (Zhang et al. 2003; Ghorbanzadeh et al. 2021). Decreasing SLA to save leaf water content is associated with the adaptation of most plants to drought stress (Delzon 2015; Wellstein et al. 2017; Dawson et al. 2022). Specific leaf area (Fig. 1) at ML and LL increased under control condition, but decreased under drought condition in *A. halimus*. The increase did not enhance *A;* maybe there were other factors that were limiting. Meanwhile, the reduction in SLA could mitigate the drought stress. In contrast to *A. halimus*, SLA increased with the decrease in light intensities under control and drought conditions, suggesting that enhancing the efficiency of light capturing was a critical factor for acclimation of *A. nummularia* to lower light intensity regardless of soil water content. Under FL condition, SLA increased in the C4 plants but not the C3 ones under drought conditions compared to the control conditions. This increase resulted from the unchanged leaf area along with the decrease in the dry weight. It seems that the C4 plants, but not the C3 ones, maintained the leaf area under both conditions for proper light capturing regardless of soil water content, resulting in low water content, *g*_*s*_ and *A*, leading to low DW. The C3 plants decreased SLA to acclimate to drought stress. This contrast response may be due to the high acclimation of the C4 plants to drought and high temperature. However, the response of the C4 species at ML and LL under drought condition is different. The increase in SLA in all species except *A. halimus* at ML and LL compared to FL under drought condition was a result of an increase in leaf area and a decrease in DW. The modification of SLA in response to drought stress is a plastic trait (Liancourt et al. 2015; Siefert et al. 2015). Increased SLA under drought condition was also detected in European sub-Mediterranean grasses, which are adapted to drought conditions (Wellstein et al. 2017 and ref. therein). From the present data, the studied plants increased leaf area at ML and LL under drought condition for efficient light capturing. Leaf area increased because of the relief of drought stress effect due to low light intensities (Zhang et al. 2003; Ghorbanzadeh et al. 2021).

Taken together under drought condition, *g*_*s*_ was controlled by soil water content and this affected *A* in *A. halimus*, but in *A. nummualria*, *g*_*s*_ was not severely affected by soil water content, but it seems that *g*_*s*_ performed better at lower light intensities and the increase in light capturing efficiency and optimizing the CO_2_ leakage enhanced the assimilation rate. In the present study, *FNR* transcript level showed a consistent trend in *A. halimus* and *A. nummularia* under control condition at 9:00 and 12:00. Meanwhile, there was no consistent pattern in FNR expression under drought condition, suggesting that each plant responded differently to the combined effect of low light and drought. The increase in FNR transcript level at ML and LL may suggest its role in alleviating the negative effect of low light, which could be the resulted oxidative stress and/or producing excess NADPH for CO_2_ fixation.

Like the C4 species, *A* and *g*_*s*_ were higher at ML and LL compared to FL, may because of lower evapotranspiration and higher leaf water content. However, unlike that under control condition, *A* was enhanced at 12:00 with the increase in *g*_*s*_, suggesting different strategies used by these C3 species in response to drought stress. Although *A* was decreased at FL, *A* was enhanced at ML and LL under drought condition. The photosynthetic rate was enhanced, maybe due to the increase in Rubisco and chlorophyll compared to that at FL. But again, this increase was not mirrored as an increase in the leaf dry biomass, but it may be invested in the biomass of all plants (stem length, number of leaves, data not shown). The increase in *A* in the C4 species was invested in the leaf and whole plant biomass. The increase in SLA may be another acclimation strategy of the C3 species to drought stress. The regulation of *FNR* transcript was not consistently regulated in *A. portulacoides* and *A. prostrata* and it did not coincide with the pattern of the photosynthetic rate. The increase in the ratio of starch/ TSS could negatively affect *A*. Starch/ TSS significantly increased in all leaves at FL in the four species compared to those at ML and LL under drought condition. The change in the size of VB and the number of VBs did not give a consistent indicator in their relationship to the assimilation rate and the transport of assimilates in the four species (Fig. 6).

## Concluding remarks

The concluding question could be: is the photosynthetic efficiency of C4 species negatively affected by lower light intensities compared to the C3 species? Or whether the C3 species acclimate better to low light intensities than the C4 ones? The C4 species maintained higher *WUE* (*A/E*) (Table 1), carboxylation efficiency (CE) (Table 2) and light use efficiency (*LUE*) (Table 3) at all light intensities than those of the C3 species under control condition, suggesting their ability to acclimate to different light intensities but under adequate soil water content. The C4 species maintained higher carboxylation efficiency at ML and LL compared to that at FL under drought condition, meaning that a combined outcome of high light intensity (FL) and water shortage could negatively affect the efficiency of the C4 species. *Atriplex halimus* showed lower *LUE* under drought condition, which could be attributed to lower *A* in this species compared to *A. nummularia,* especially at 12:00, suggesting that soil water content could be a main limiting factor in *A. halimus.* The rise of photosynthesis and efficient use of water in C4 plants could be attributed to the environmental variables (Osborne and Sack 2012; Griffiths et al. 2013). However, these could be mainly dependent on the species, according to the present results.

**Table 1:**
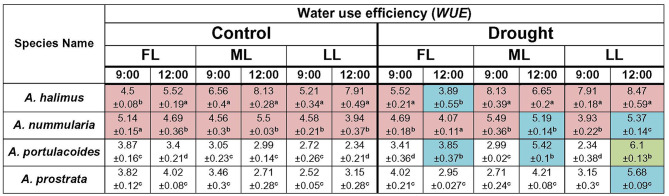
Diurnal response of water use efficiency (photosynthetic rate, *A* / transpiration rate, *E*) of the four species of *Atriplex* at different light intensities under control and drought conditions. FL: full light intensity, ML: medium light intensity and LL: low light intensity. The brown colour represents the superior response of the C4 species over the C3 ones. The green colour represents the superior response of the C3 species over the C4 ones. The blue colour represents the equal response of the C3 and C4 species. Data are mean ± SE. Data labeled with different letters are significantly different at *P* ≤ 0.05

**Table 2:**
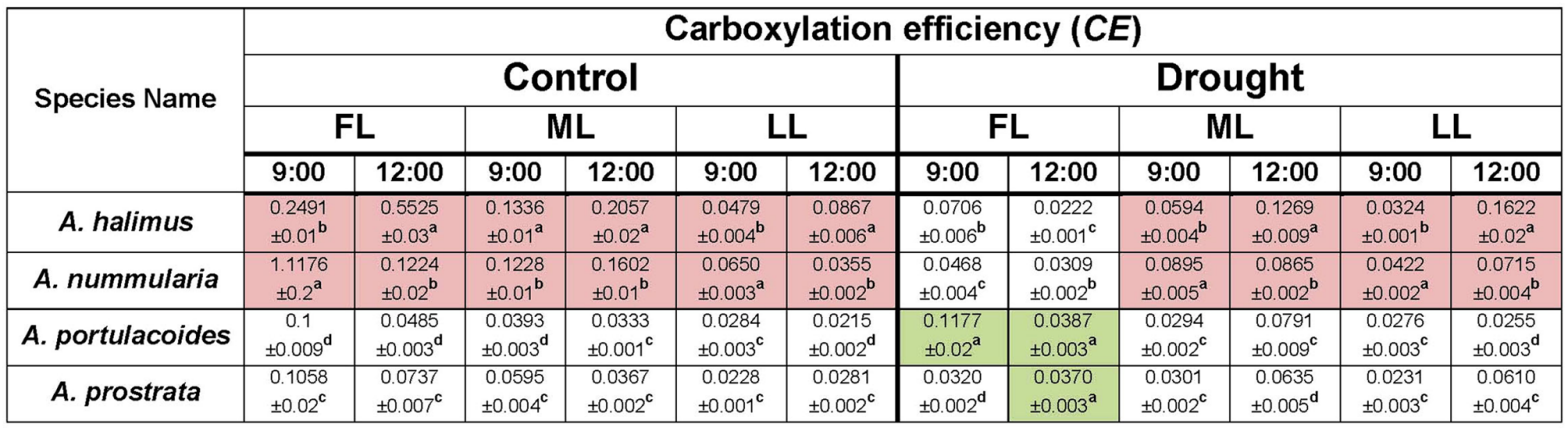
Diunal response of carboxylation efficiency (photosynthetic rate, *A* / internal CO_2_, *C*_*i*_) of the four species of *Atriplex* at different light intensities under control and drought conditions. FL: full light intensity, ML: medium light intensity and LL: low light intensity. The brown colour represents the superior response of the C4 species over the C3 ones. The green colour represents the superior response of the C3 species over the C4 ones. Data are mean ± SE. Data labeled with different letters are significantly different at  *P* ≤ 0.05

**Table 3:**
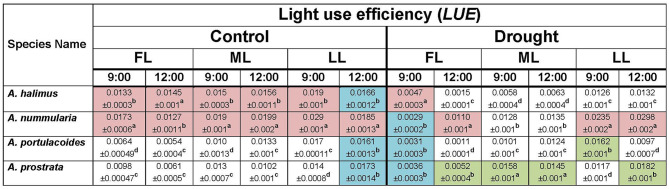
Diurnal response of light use efficiency (photosynthetic rate, *A* / quantum leaf, Q) of the four species of *Atriplex* at different light intensities under control and drought conditions. FL: full light intensity, ML: medium light intensity and LL: low light intensity. The brown colour represents the superior response of the C4 species over the C3 ones. The green colour represents the superior response of the C3 species over the C4 ones. The blue colour represents the equal response of the C3 and C4 species. Data are mean ± SE.   Data labeled with different letters are significantly different at *P* ≤ 0.05

Based on the biomass results, *LUE* of the C4 species was higher than that of the C3 ones. However, *WUE*_*i*_ was higher in the C4 species under control and drought conditions at most points, which suggests that C4 species could be characterized by controlled and regulated relationships between *A*, *gs* and *C*_*i*_. The reduction in CO_2_ during the exposure of high light intensity was dramatically affecting the assimilation rate of C3 species, as suggested by Kluge et al. (2015). Jones (1985) suggested that removal of stomatal limitation increased the photosynthetic rate by only 10–20% in C3 species and even less in C4 species. The decrease in carboxylation efficiency and *WUE*_*i*_ in the C3, especially at ML and LL, could be attributed to the increase in *g*_*s*_ and *C*_*i*_, which was not mirrored as an increase in *A* or biomass. In the present study, the C4 species, especially *A. nummularia*, efficiently use the available resources and regulate *g*_*s*_ and *C*_*i*_ to achieve maximum *A* and biomass. Moreover, the C4 but not the C3 species could adjust *A* according to *g*_*s*_, and it could be expected that the C4 species could have efficiently acclimated photosystems to low light intensities compared to the C3 species. Therefore, the C4 species could acclimate more efficiently to low light intensity than the C3 ones, but this efficient acclimation could be dependent on species and environmental conditions but not on the sub-biochemical type. Understanding the response of the C4 *Atriplex* species to low light intensity under different soil water regimes could add valuable routes to the programme of the engineering of C4 photosynthesis into C3 plants. The present study could provide basic points that can be used as starting points for further studies. Therefore, further investigations with advanced techniques in this context are required for full understanding.

## Electronic supplementary material

Below is the link to the electronic supplementary material.

Supplementary Material 1

## Data Availability

No datasets were generated or analysed during the current study.
