# Supplementary material for: Diurnal light fitness of the C3 and C4 species from the genus Atriplex under control and drought conditions
Source: Photosynth Res. 2025 Jun 11;163(3):35. doi: 10.1007/s11120-025-01154-5 (PMC12158838; doi:10.1007/s11120-025-01154-5)
Supplement: Supplementary file 1 — Supplementary Material 1 [file 11120_2025_1154_MOESM1_ESM.pdf]

Diurnal light fitness of the C3 and C4 species from the genus *Atriplex* under control and drought conditions

Reham M. Nada[1,*], Abdel Hamid A. Khedr[1], Mamdouh S. Serag[1], Nesma R. El-Qashlan[1] and Gaber M. Abogadallah[1]

[1] Department of Botany and Microbiology, Faculty of Science, Damietta University, New Damietta 34517, Egypt

*Author for correspondence: Email: rnada@du.edu.eg

Tel: 00201090175013

ORCID ID: https://orcid.org/0000-0002-4552-5383

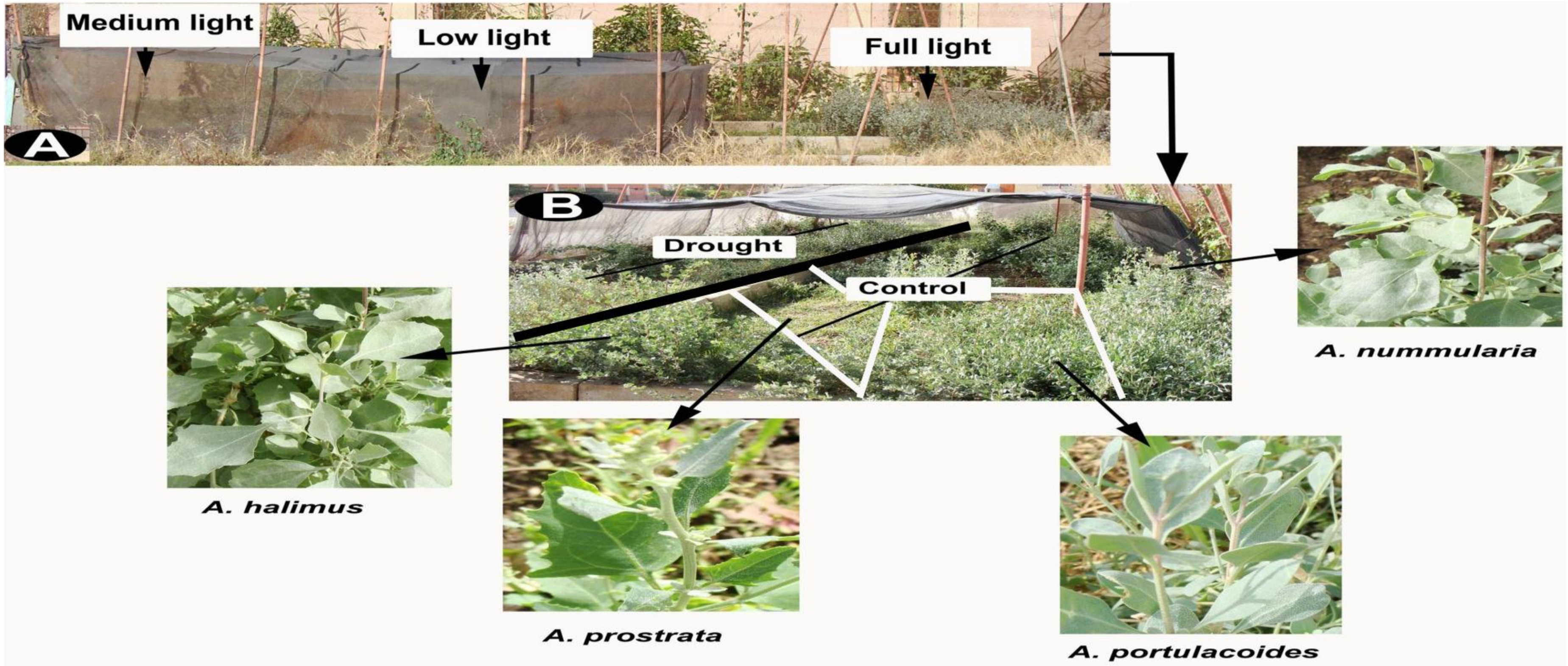

Figure S1. *Atriplex* species grown in the green house, Botany and Microbiology Department, Faculty of Science, Damietta University. A: Side view of the green house. B: is an internal view of the green house showing the design of the experiment. Bushes were divided into two groups (control and drought). Each group (control or drought) was divided into three categories; the first was exposed to full sun-light, the second was exposed to medium light and the third was exposed to low light intensity as shown in A and B. the greenhouse was divided into blocks: a block for each species under each treatment.

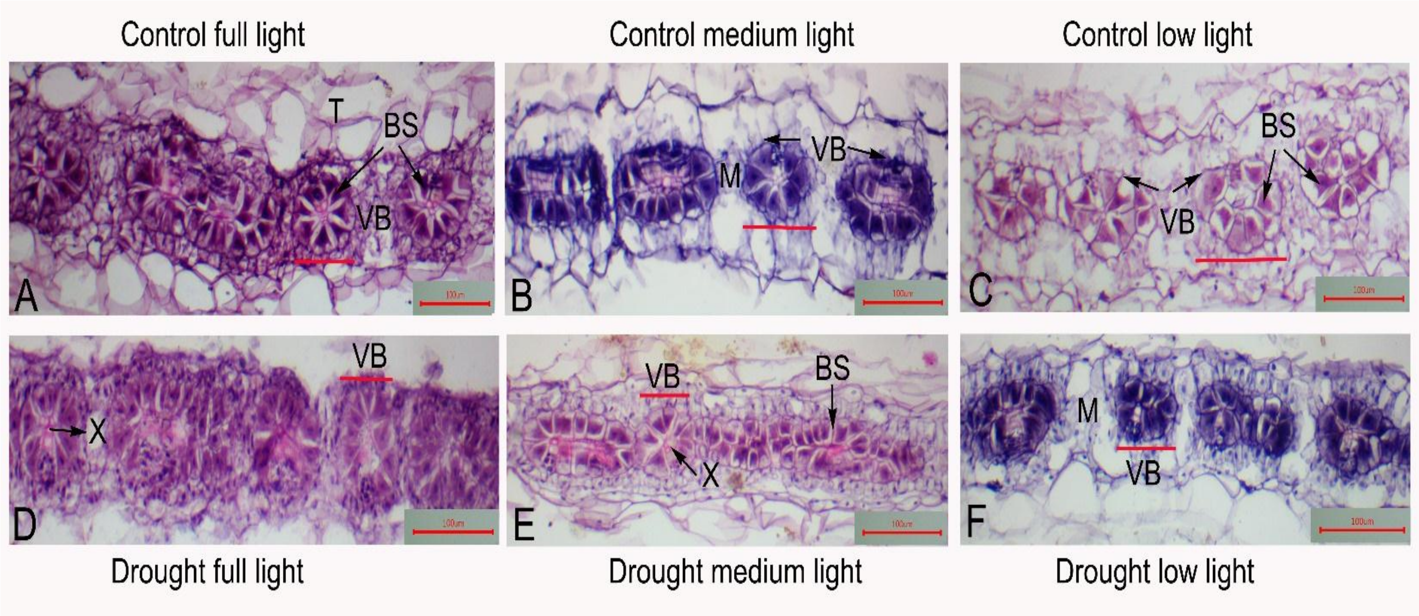

Figure S2. Transverse section of *A. halimus* leaf under control and drought conditions with different light intensities; VB is the vascular bundle, BS is the bundle sheath, T is the trichome, M is the mesophyll and X is the xylem

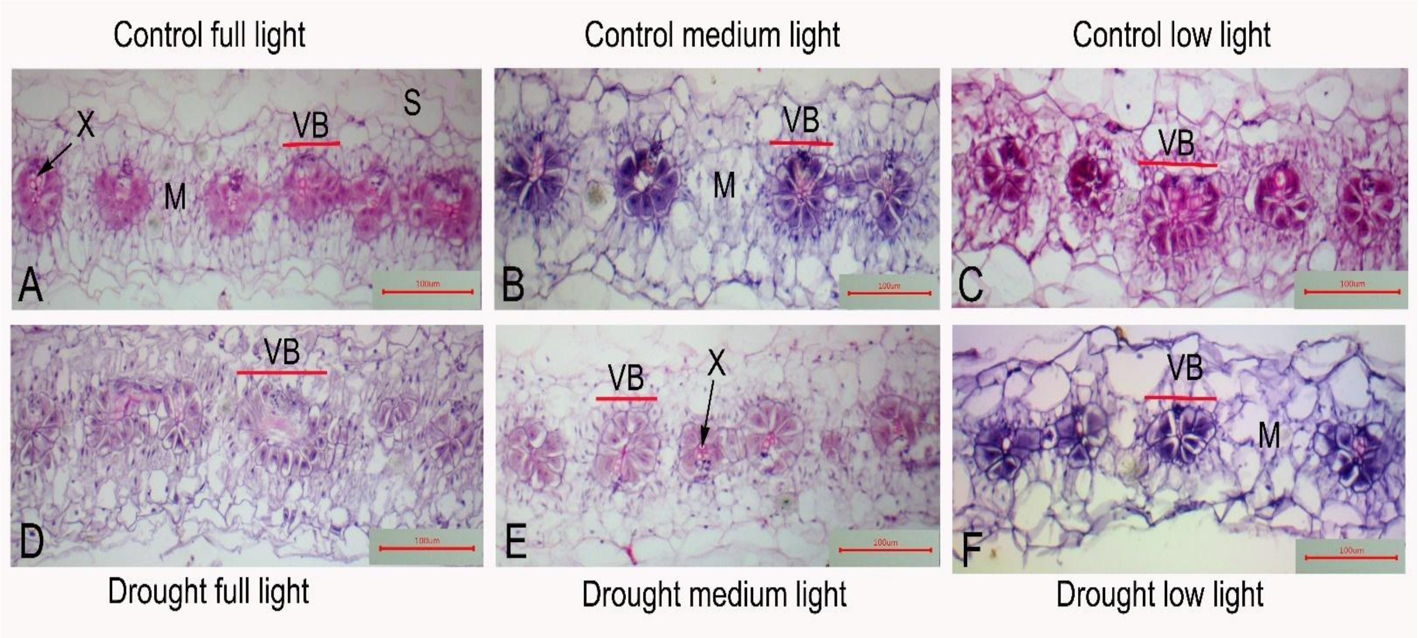

Figure S3. Transverse section of *A. nummularia* leaf under control and drought conditions with different light intensities; VB is the vascular bundle, M is the mesophyll, S is the salt gland and X is the xylem.

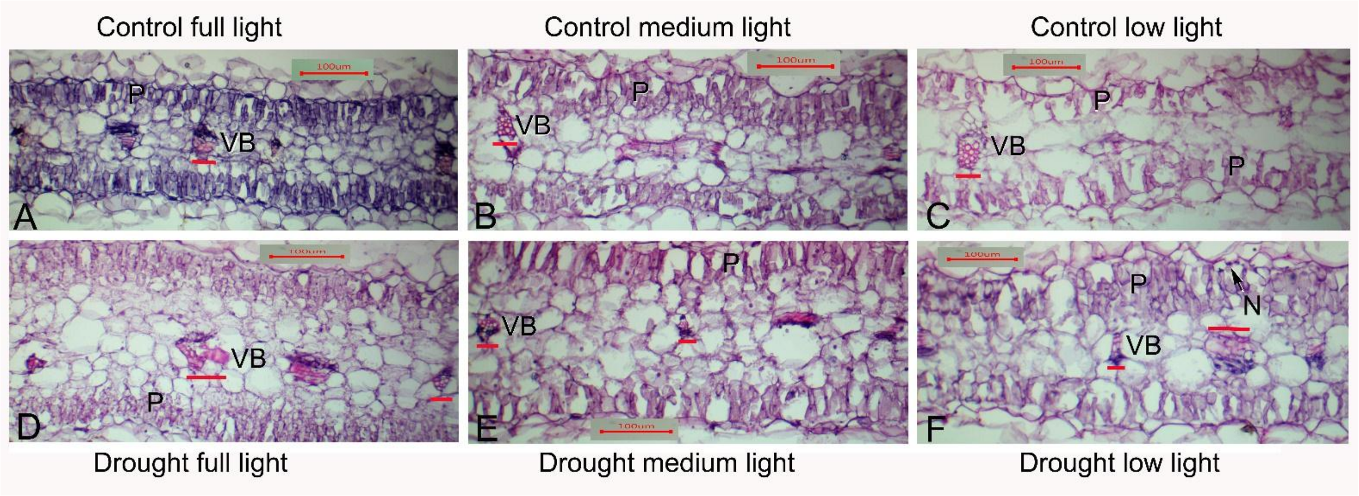

Figure S4. Transverse section of *A. portulacoides* leaf under control and drought conditions with different light intensities; VB is the vascular bundle, P is the palisade tissue and N is the nucleus.

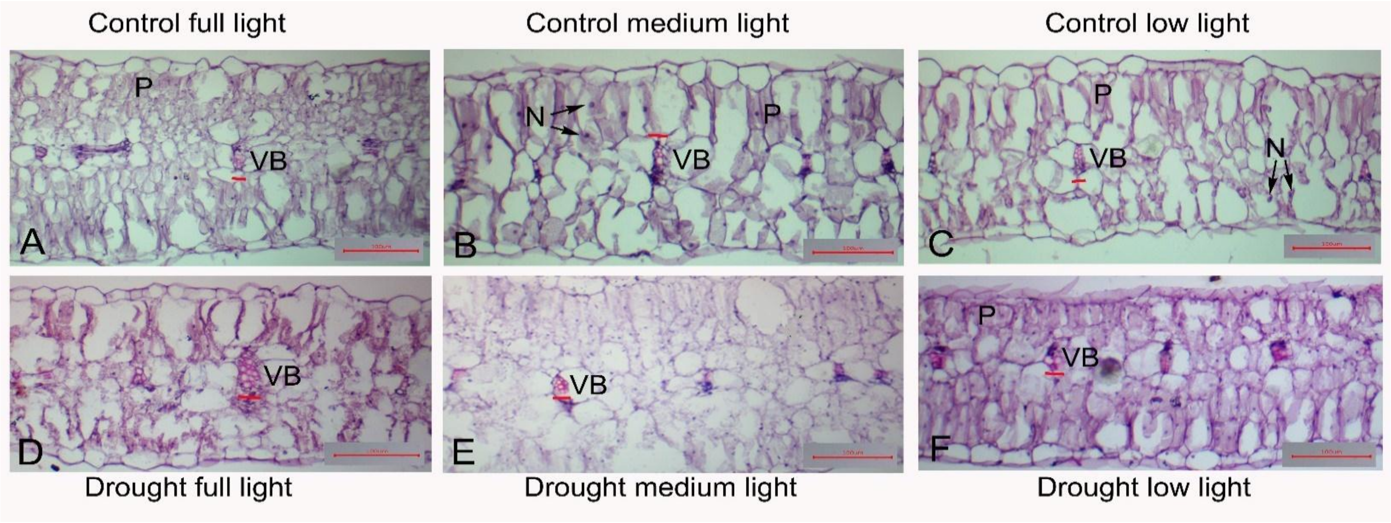

Figure S5. Transverse section of *A. prostrata* leaf under control and drought conditions with different light intensities; VB is the vascular bundle, P is the palisade tissue and N is the nucleus.

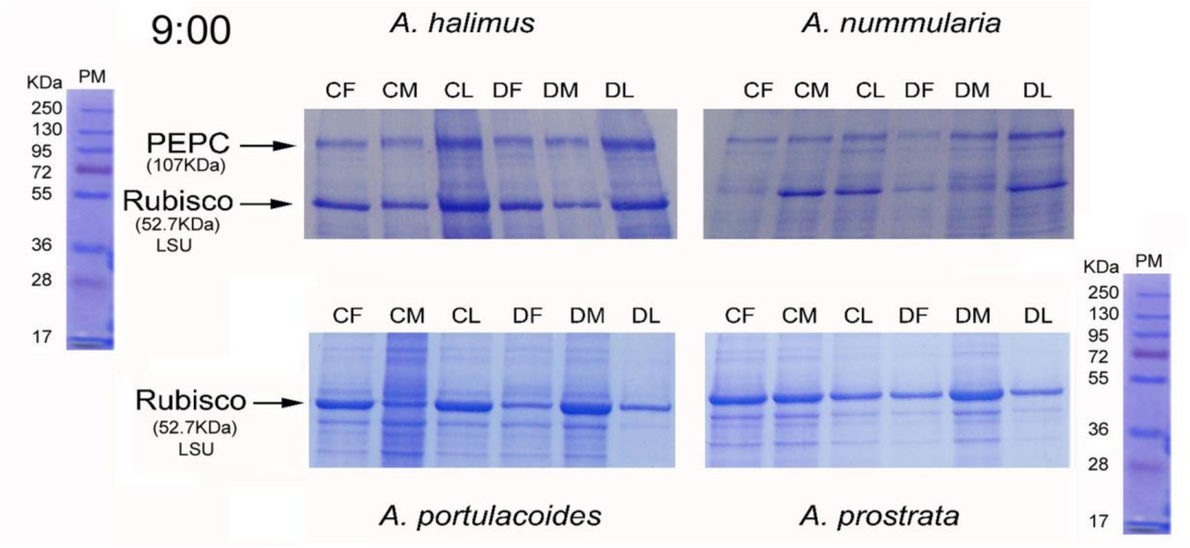

Figure S6. Rubisco and PEPC protein of *Atriplex* species at 9:00 under different light intensities under control and drought conditions.SDS-PAGE for leaf protein loaded on the basis of unit mg of leaf protein. CF is control full light, CM is control medium light, CL is control low light, DF is drought full light, DM is drought medium light and DL is drought low light. PM: protein marker. LSU: Rubisco large subunit.

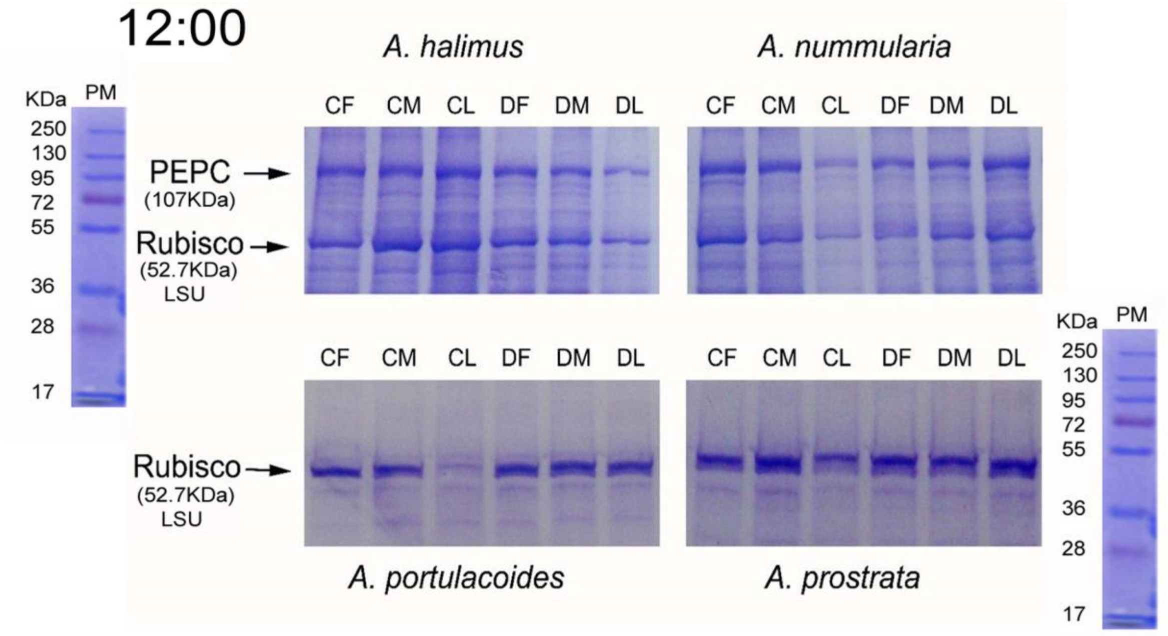

Figure S7. Rubisco and PEPC protein of *Atriplex* species at 12:00 under different light intensities under control and drought conditions.SDS-PAGE for leaf protein loaded on the basis of unit mg of leaf protein. CF is control full light, CM is control medium light, CL is control low light, DF is drought full light, DM is drought medium light and DL is drought low light. PM: protein marker. LSU: Rubisco large subunit.

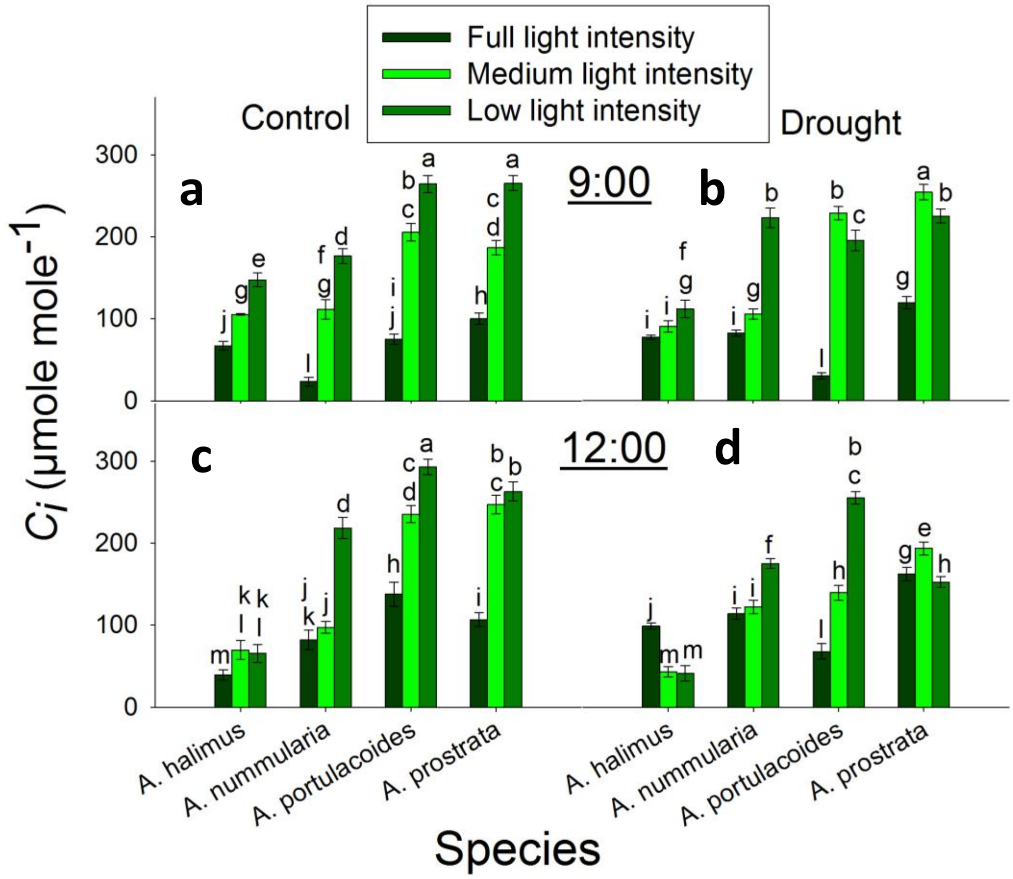

Figure S8. Diurnal response of internal CO_2 (*Ci*) in the four species of *Atriplex* to different light intensities under control and drought conditions. a and b at 9:00 and c and d at 12:00. Data are mean ± SE. Bars labelled with different letters are significantly different at $P \leq 0.05$.

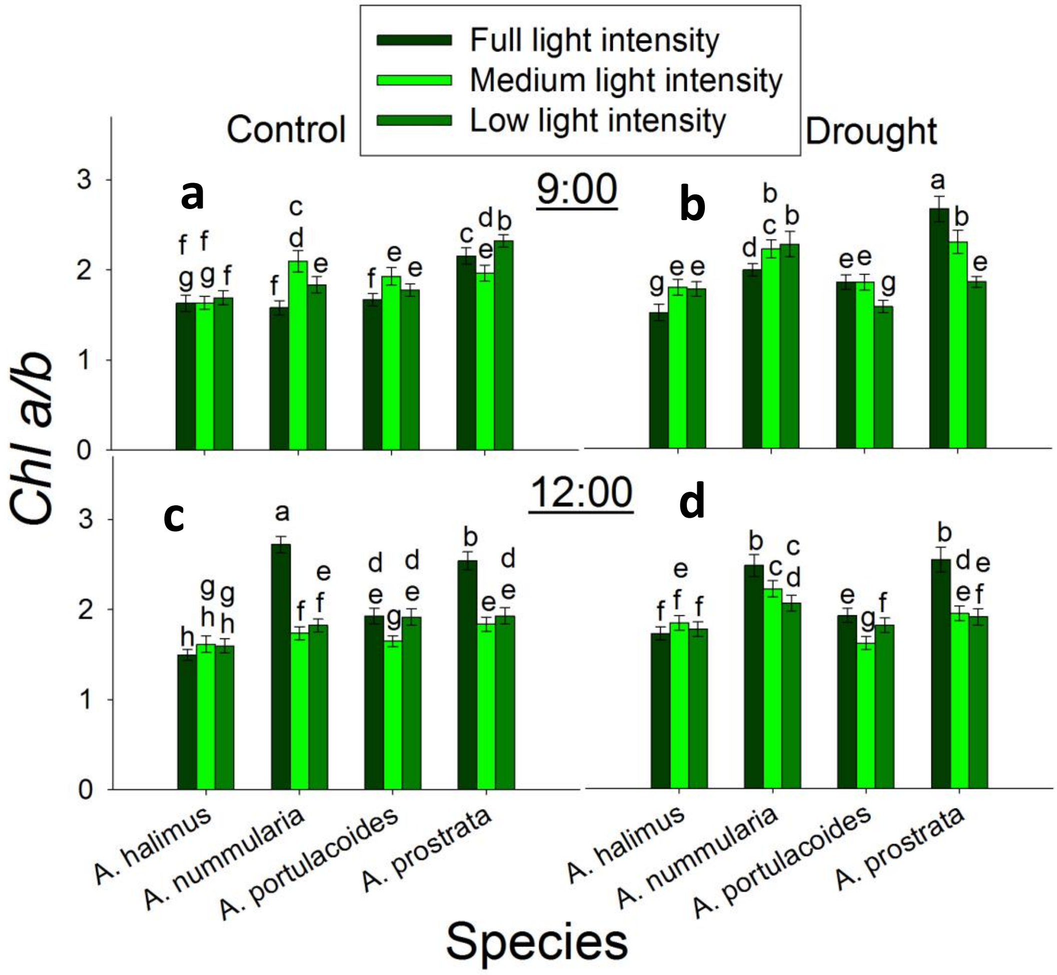

Figure S9. Diurnal response of Chl a / Chl b ration in the four species of *Atriplex* to different light intensities under control and drought conditions. a and b at 9:00 and c and d at 12:00. Data are mean ± SE. Bars labelled with different letters are significantly different at $P \leq 0.05$.

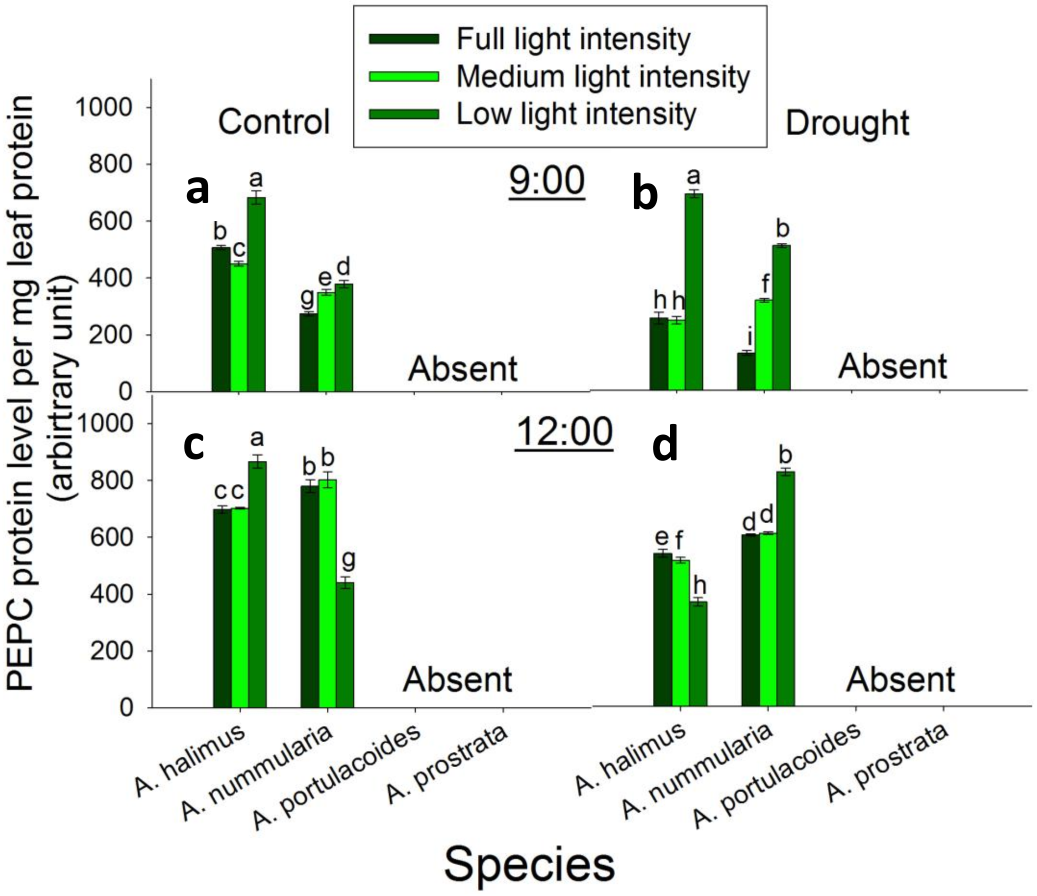

Figure S10. Diurnal response of PEPC protein level per unit leaf soluble protein in the four species of *Atriplex* to different light intensities under control and drought conditions. a and b at 9:00 and c and d at 12:00. Data are mean ± SE. Bars labelled with different letters are significantly different at $P \leq 0.05$.

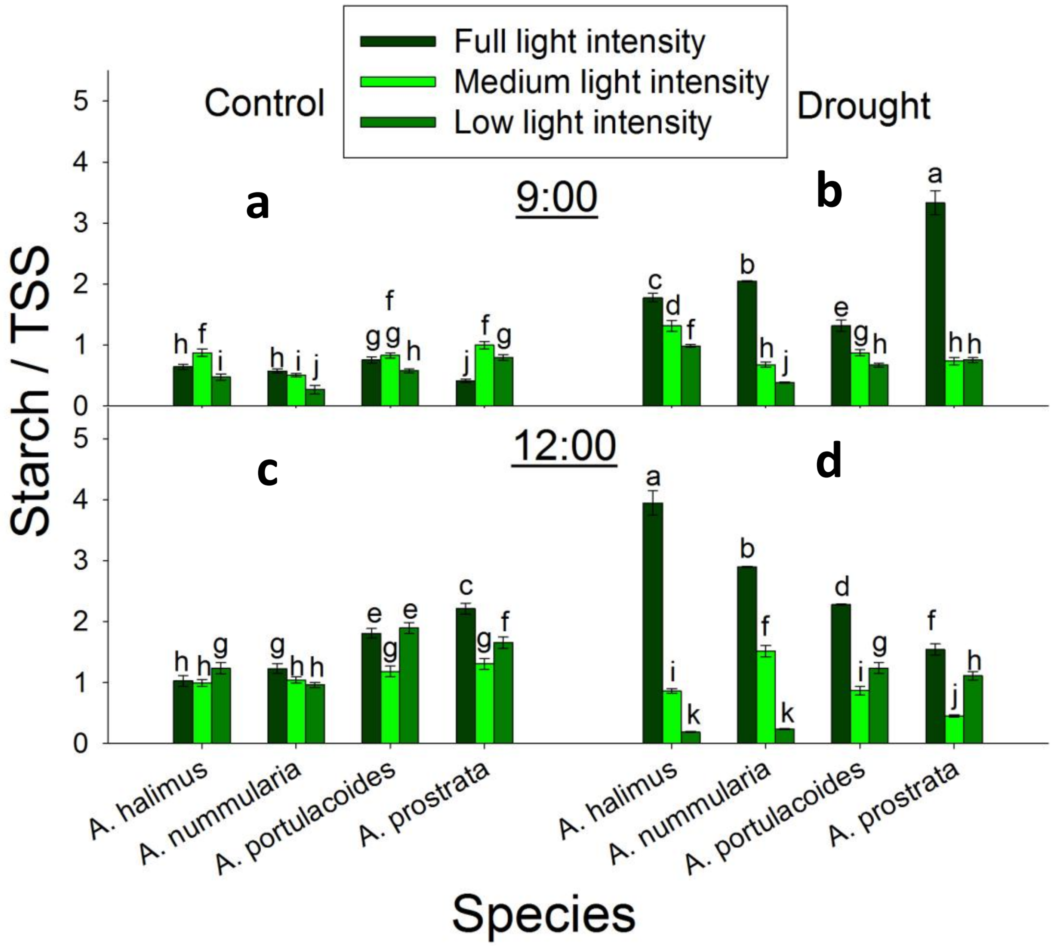

Figure S11. Diurnal response of starch/ total soluble sugars (TSS) ratio in the four species of *Atriplex* to different light intensities under control and drought conditions. a and b at 9:00 and c and d at 12:00. Data are mean ± SE. Bars labelled with different letters are significantly different at $P \leq 0.05$.

Table S1. Primers used to amplify FNR and AOX genes in the present study.

Primer Name	Forward primer	Reverse primer
FNR	GGNGARACNTGGCAYATGG	TANACYTCNACRTTCCAYTG
AOX	GNTAYGGNTGYMGNGCNATG	CKRTGRTGNGCYTCRTCNGC

Table S2. Diurnal response of photosynthetic efficiency (photosynthetic rate, *A* / Rubisco) of the four species of *Atriplex* at different light intensities under control and drought conditions. FL: Full light intensity, ML: Medium light intensity and LL: Low light intensity. The brown colour represents the superior response of the C4 species over the C3 ones. The green colour represents the superior response of the C3 species over the C4 ones. The blue colour represents the equal response of the C3 and C4 species. Data are mean ± SE. n= 3. Data labeled with different letters are significantly different at $P \leq 0.05$.

Species Name	Photosynthetic efficiency (*A*/ Rubisco)											
	Control						Drought					
	FL		ML		LL		FL		ML		LL	
	9:00	12:00	9:00	12:00	9:00	12:00	9:00	12:00	9:00	12:00	9:00	12:00
A. halimus	0.0144 ±0.001^{b}	0.031 ±0.003^{b}	0.0146 ±0.004^{c}	0.0146 ±0.002^{b}	0.0036 ±0.0004^{b}	0.0057 ±0.0001^{d}	0.0048 ±0.0002^{b}	0.0042 ±0.0001^{b}	0.0083 ±0.0004^{b}	0.0064 ±0.0009^{b}	0.0021 ±0.0001^{c}	0.012 ±0.0003^{b}
A. nummularia	0.2445 ±0.003^{a}	0.0149 ±0.003^{a}	0.0165 ±0.005^{b}	0.0299 ±0.002^{a}	0.0169 ±0.003^{a}	0.0234 ±0.001^{b}	0.0217 ±0.002^{a}	0.0120 ±0.002^{a}	0.0570 ±0.001^{a}	0.0175 ±0.002^{a}	0.0111 ±0.002^{a}	0.0165 ±0.001^{a}
A. portulacoides	0.0045 ±0.0006^{d}	0.0056 ±0.0009^{d}	0.0268 ±0.006^{a}	0.007 ±0.0001^{c}	0.004 ±0.0003^{b}	0.0299 ±0.0002^{a}	0.0043 ±0.0003^{c}	0.0016 ±0.0003^{d}	0.0033 ±0.0002^{c}	0.0065 ±0.0009^{b}	0.0053 ±0.0003^{b}	0.0038 ±0.0003^{d}
A. prostrata	0.0061 ±0.0005^{c}	0.0061 ±0.0007^{c}	0.0068 ±0.002^{d}	0.0045 ±0.0001^{d}	0.0044 ±0.0001^{b}	0.0088 ±0.0002^{c}	0.0034 ±0.0006^{d}	0.0033 ±0.0003^{c}	0.0039 ±0.002^{c}	0.0067 ±0.0005^{b}	0.0053 ±0.0003^{b}	0.0049 ±0.0004^{c}

Table S3. Diurnal response of intrinsic water use efficiency (photosynthetic rate, *A* / stomatal conductance, g_s) of the four species of *Atriplex* at different light intensities under control and drought conditions. FL: Full light intensity, ML: Medium light intensity and LL: Low light intensity. The brown colour represents the superior response of the C4 species over the C3 ones. The green colour represents the superior response of the C3 species over the C4 ones. The blue colour represents the equal response of the C3 and C4 species. Data are mean ± SE. n= 3. Data labeled with different letters are significantly different at $P \leq 0.05$.

Species Name	Intrinsic water use efficiency (*WUE$_i$*)											
	Control						Drought					
	FL		ML		LL		FL		ML		LL	
	9:00	12:00	9:00	12:00	9:00	12:00	9:00	12:00	9:00	12:00	9:00	12:00
A. halimus	172.6 ±1.7^b	176.2 ±13^a	234.4 ±6.7^a	226.4 ±7.1^a	151.1 ±14.3^a	200.2 ±11^a	272 ±19.5^a	220 ±18.8^a	200 ±15^a	229.2 ±13^a	121 ±17.5^a	164.7 ±7.6^a
A. nummularia	177.8 ±5.8^b	167.3 ±16^b	146.6 ±15^b	155.9 ±4^b	110.9 ±6.6^b	96.9 ±9^b	143.8 ±10^c	176 ±6.8^b	157 ±13^b	151.2 ±11.6^b	93.9 ±8.4^b	107.3 ±4.9^d
A. portulacoides	204.44 ±11^a	154 ±11^c	96.9 ±11^d	78.4 ±4.6^c	62.6 ±4.1^c	46.03 ±5.5^d	151.5 ±11^b	131 ±12^d	87.5 ±4.5^c	138 ±1.6^c	84.9 ±7.6^c	139.2 ±3.1^b
A. prostrata	167.1 ±11^c	168.3 ±6.6^b	101 ±6^c	64.7 ±8.4^d	62.5 ±1.2^c	67.1 ±8.6^c	127 ±4.8^d	157.6 ±3.8^c	84.9 ±8^c	97 ±4.1^d	97.5 ±9.1^b	121.3 ±1.1^c
